# Investigation of growth dynamics of carbon nanotubes

**DOI:** 10.3762/bjnano.8.85

**Published:** 2017-04-11

**Authors:** Marianna V Kharlamova

**Affiliations:** 1 Faculty of Physics, University of Vienna, Strudlhofgasse 4, 1090 Vienna, Austria

**Keywords:** activation energy, carbon nanotube, growth dynamics, growth rate, synthesis

## Abstract

The synthesis of single-walled carbon nanotubes (SWCNTs) with defined properties is required for both fundamental investigations and practical applications. The revealing and thorough understanding of the growth mechanism of SWCNTs is the key to the synthesis of nanotubes with required properties. This paper reviews the current status of the research on the investigation of growth dynamics of carbon nanotubes. The review starts with the consideration of the peculiarities of the growth mechanism of carbon nanotubes. The physical and chemical states of the catalyst during the nanotube growth are discussed. The chirality selective growth of nanotubes is described. The main part of the review is dedicated to the analysis and systematization of the reported results on the investigation of growth dynamics of nanotubes. The studies on the revealing of the dependence of the growth rate of nanotubes on the synthesis parameters are reviewed. The correlation between the lifetime of catalyst and growth rate of nanotubes is discussed. The reports on the calculation of the activation energy of the nanotube growth are summarized. Finally, the growth properties of inner tubes inside SWCNTs are considered.

## Review

### Introduction

Single-walled carbon nanotubes (SWCNTs) discovered in 1993 [1–2] possess extraordinary physical, chemical and mechanical properties [3]. They are unique nanoscale objects, because their electronic structure (metallic or semiconducting) is solely dependent on the atomic structure [3–4]. Since the discovery of SWCNTs, attempts of many researchers have been aimed at developing the methods of their efficient synthesis. During last years, significant progress was made in this field. The arc-discharge, laser ablation and chemical vapor deposition (CVD) methods were optimized for the synthesis of SWCNTs in a high yield [5–6]. Synthesis parameters can be varied in a broad range, which leads to the production of SWCNTs with defined morphology and high purity. Although selective synthesis of SWCNTs with certain conductivity type and structure was attempted [7–8], typical as-synthesized samples consist of a mixture of metallic and semiconducting SWCNTs [6]. This causes inhomogeneity of their properties.

The synthesis of SWCNTs with defined properties is required for both fundamental investigations and practical applications. Despite the fact that the use of SWCNTs in the fields of nanoelectronics [9–12], thin-film flexible electronics [13–14] and bioelectronics [15] was already demonstrated, many applications of SWCNTs were not yet realized. The revealing and thorough understanding of the growth mechanism of SWCNTs is the key to the synthesis of nanotubes with required properties.

The aim of this manuscript is to deliver a comprehensive review of the current status of the research on the investigation of growth dynamics of carbon nanotubes. In the first part of the review, the peculiarities of the growth mechanism of carbon nanotubes are discussed. The well-accepted growth models of nanotubes are highlighted. Among them are the vapor–liquid–solid and vapor–solid–solid models, the tip- and base-growth models as well as the tangential and perpendicular growth modes. The physical and chemical states of the catalyst during the nanotube growth are considered. The chirality selective growth of nanotubes is described. The main part of the review is dedicated to the analysis and systematization of reported results on the investigation of growth dynamics of nanotubes. The models suggested for the description of growth dynamics of nanotubes are presented. The studies on the revealing of the dependence of the growth rate of nanotubes on the synthesis parameters (the pressure of carbon precursor, size and chemical nature of catalyst particle, synthesis temperature) are reviewed. The correlation between the lifetime of catalyst and growth rate of nanotubes is discussed. The reports on the calculation of the activation energy of the nanotube growth are summarized. Finally, the growth properties of inner tubes inside SWCNTs filled with fullerene and organometallic molecules are considered.

### Synthesis of carbon nanotubes

The SWCNTs can be synthesized by the arc-discharge, laser ablation and chemical vapour deposition (CVD) techniques. A detailed overview of these synthesis procedures can be found in previous reviews [5–6,16–17].

The synthesis methods of SWCNTs include also the growth of tubes inside the outer SWCNTs. The inner tubes can be formed inside SWCNTs filled with molecules of fullerenes, metallocenes, acetylacetonates and other precursors, as described in detail in [18].

### Growth mechanism of carbon nanotubes

Although the synthesis of nanotubes with controlled properties can be performed in the CVD process, the growth mechanism of nanotubes is not completely understood and is still debated.

#### Nanotube growth in the CVD process

**Vapor-liquid-solid and vapor-solid-solid growth models.** In the 1970s, Baker with co-authors suggested in [19–21] that the growth of carbon filaments occurred by the vapor–liquid–solid (VLS) model, which was previously developed by Wagner and Ellis to explain the growth of silicon whiskers [22]. In the growth process of Si whiskers, the initial condition was the formation of a liquid droplet of the alloy of Si with Au impurity on a Si wafer. The liquid Au–Si alloy acted as a preferred sink for the deposition of Si atoms from the vapor that was obtained as a result of the thermally-induced decomposition of gaseous SiCl_4_. As soon as the liquid alloy particle was supersaturated, the growth of the whisker started. It occurred by the precipitation of Si atoms from the droplet at the interface between solid Si and liquid alloy. As a result, the alloy droplet was displaced from the Si substrate crystal to the tip of the growing whisker [22]. Thus, the VLS model of Wagner and Ellis implied two hypotheses: (i) the particle was liquid and (ii) the diffusion of reactant atoms occurred through the bulk of the particle.

The use of the VLS model for the growth of carbon filaments and nanotubes received massive support, because the activation energies of the growth calculated by Baker with co-authors were in good agreement with the activation barriers of the bulk carbon diffusion through the corresponding metals, which was defined as the growth rate-limiting process [19–21]. However, Baker with co-authors compared the calculated activation energies with those of the bulk carbon diffusion through metals in the solid state and not in the liquid state. Therefore, their results supported only the bulk diffusion hypothesis of the VLS model and contradicted the liquid particle hypothesis. The employment of the term “VLS model” for the description of the growth mechanism on solid catalyst particles is often misleading. The considered hypothesis of the VLS model should be preferably specified [23].

The VLS model in its classical interpretation, which obeys two hypotheses of Wagner and Ellis, was used to explain the growth of carbon filaments on liquid catalysts [24–26]. Later on, the VLS model was applied to describe the growth of MWCNTs [27] and SWCNTs [28–29] on liquid-metal particles. The atomic-level description of the VLS growth process of SWCNTs was performed by molecular dynamics simulations [29–32].

There are three different steps in the VLS growth mechanism of carbon filaments and nanotubes. In the first step, atomic carbon is provided on the surface of a hot metallic particle by dissociation of adsorbed molecules. In the second step, carbon dissolves into the bulk of the catalyst particle. A liquid carbon–metal solution is formed. Carbon diffuses through the liquid particle. In the third step, when the carbon–metal solution becomes saturated the dissolved carbon precipitates in the form of cylindrical or tubular networks of sp^2^ carbon [33–34].

The motor for the directed diffusion from the dissociating surface to the precipitating surface through the bulk of the catalyst particle was actively debated. Originally a temperature gradient across the catalyst particle was suggested as a driving factor for the bulk diffusion by Baker and coworkers [19]. The temperature gradient would be maintained by the exothermic catalytic decomposition of the precursor molecules and the endothermic precipitation of carbon at opposing face of the catalyst particle. However, these requirements are not met in the case of endothermic decomposition of for instance alkanes [33,35–37] and the hypothesis of a temperature gradient is further challenged for small nm sized particles, which can grow single-walled carbon nanotubes. It is unlikely to play an important role in the growth of SWCNTs, because small catalytic particles have a high thermal conductivity and therefore the temperature gradient would lead to an unphysically large heat flow [30,32]. Molecular dynamics simulations performed in [30,32] showed that the carbon concentration gradient within the catalytic particle is important for the VLS growth of SWCNTs, whereas the temperature gradient is not necessary. Thermodynamic calculations conducted in [38] also indicated that the nanotube growth is mainly driven by the carbon concentration gradient in the catalytic particle.

In the late 1970s, Oberlin with co-authors suggested an alternative mechanism to the VLS process for the description of the growth of hollow carbon filaments [39]. It implied the carbon diffusion on the surface of the metallic catalytic particle and not in its bulk. Later on, this growth mechanism was used by other authors to explain the formation process of carbon nanofibers [40–42] and nanotubes [43]. In [42], Hofmann with co-authors provided the surface diffusion model for the growth of carbon fibers on metallic catalysts on the basis of the fact that the calculated activation energies of the growth were much lower than those of the bulk carbon diffusion in the metal. They suggested that the surface carbon diffusion on the catalytic particle was also the rate-limiting step of the growth. The authors of [43] applied the surface diffusion model to explain fast growth rates of SWCNTs in the thermal CVD process at temperatures as low as 600 °C. In [41], Helveg with co-authors performed the first time-resolved in situ HRTEM studies on the formation of carbon nanofibers on nickel nanoparticles and suggested the growth mechanism involving the surface diffusion. They observed the movement of atoms on the surface of the crystalline nickel cluster and change of its shape during the growth process. It was concluded that the surface transport of carbon atoms was the growth rate-limiting process. The surface diffusion mechanism of the growth of carbon nanofibers and nanotubes on metallic catalysts was also revealed by theoretical methods [41–42,44]. In [44], Raty with co-authors reported ab initio molecular dynamics simulations of the formation of SWCNTs on metallic nanoparticles. They showed that the SWCNT growth on ≈1 nm Fe particles occurred without the diffusion of carbon atoms into the bulk of the catalyst. The carbon diffusion on the surface of the particle was much faster than the bulk diffusion.

Because the surface diffusion mechanism is observed for the growth of carbon nanofibers and nanotubes on solid catalysts, it is often called the vapor–solid–solid (VSS) mechanism, by the analogy to the VLS mechanism. Particularly, this term is used in several reviews [33–34,45]. The authors of [33] describe the VSS mechanism by three steps, including the dissociation of gaseous carbon precursor on the surface of the catalytic particle, the surface diffusion of carbon atoms on the solid particle and the precipitation of carbon in the form of nanotubes. The similarity of the terms “VLS” and “VSS” and different types of the carbon diffusion involved in these growth mechanisms may be misleading. Moreover, there is no special term for the growth mechanism that includes the bulk carbon diffusion through the solid catalytic particle. Preferably, one should clearly mention the type of carbon diffusion while using the term “VSS model” for the growth of nanotubes.

The formation of nanotubes on nonmetallic catalysts has peculiarities as compared to the growth on metals [46–49]. Catalytic nanoparticles of diamond [47], zirconia [48] and silica [46,49] have negligibly small bulk solubility of carbon, and it is therefore unlikely that the bulk carbon diffusion contributes to the nanotube growth. It was reported that the formation of SWCNTs on solid nonmetallic catalysts is promoted by the surface diffusion of carbon, suggesting the VSS growth mechanism [46–49].

**Figure 1:**
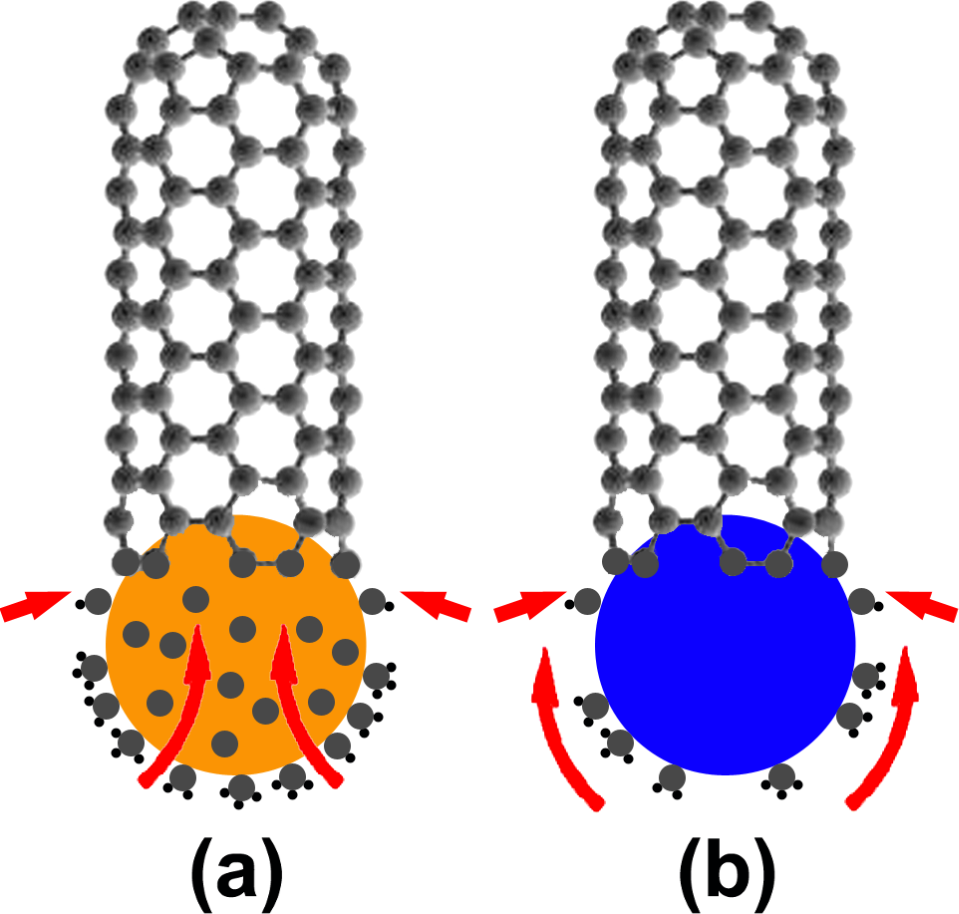
The comparison of the VLS mechanism of the SWCNT growth on the metallic catalytic particle (a) and the VSS mechanism of the growth on the SiO_2_ nanoparticle (b). In the VLS growth model (a), a gaseous carbon precursor adsorbs and dissociates on the surface of the metallic catalytic particle (orange ball). The obtained carbon atoms (grey balls) get dissolved into the metal and diffuse through the bulk of the liquid particle (as shown by large red arrows). After reaching the supersaturation, the dissolved carbon precipitates at the rear side of the particle to form a nanotube (as shown by small red arrows). In the VSS growth model (b), after the dissociation of a carbon precursor, carbon atoms diffuse on the surface of the solid catalytic particle (blue ball) and precipitate in the form of a nanotube. Figure is redrawn with modifications from [46].

Figure 1 compares the classical VLS mechanism of the SWCNT growth on the metallic catalytic particle and the VSS mechanism of the growth on the SiO_2_ nanoparticle [46].

**Physical state of catalyst.** The diameter of metallic catalytic particles for the production of nanotubes varies from one to tens of nanometers. Decreasing the diameter of the metallic particles to the nanometer scale leads to an increase in the ratio of surface atoms to internal atoms [50]. The surface atoms are electronically and coordinatively unsaturated. This leads to changed physical and chemical properties of nanoparticles in comparison to the bulk metal, for example, lower melting temperature and higher carbon solubility [50].

**Figure 2:**
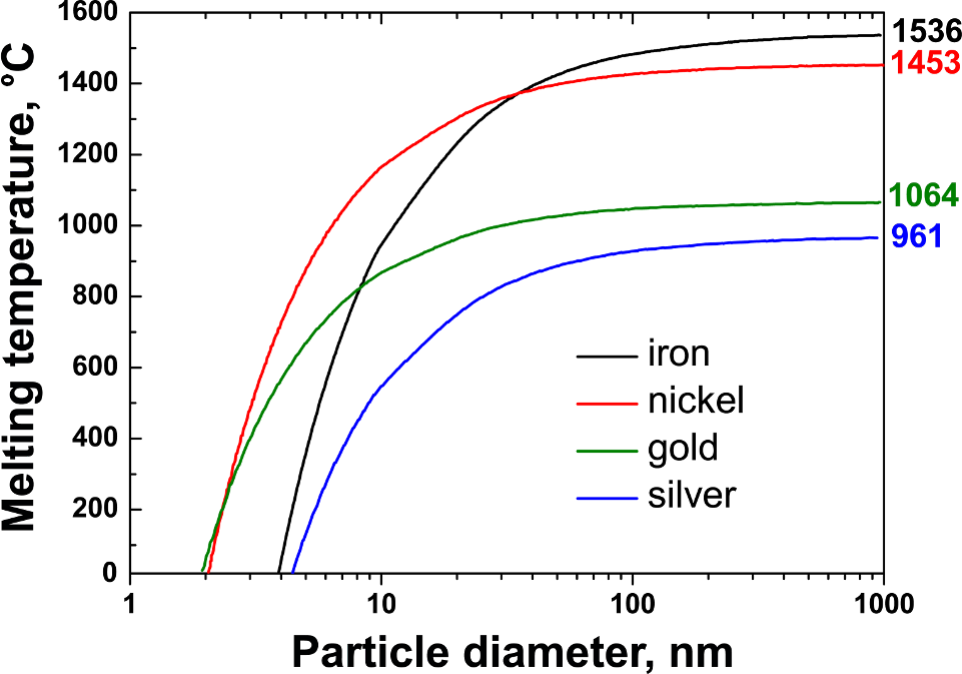
The melting temperature of iron, nickel, gold and silver particles as a function of the diameter. The data are replotted from [50].

The melting temperature of metallic catalytic particles is lowered by two effects. Firstly, the melting temperature of the particle (*T*_p_) with the radius *r* is decreased by the Gibbs–Thomson effect by the equation:

[1]
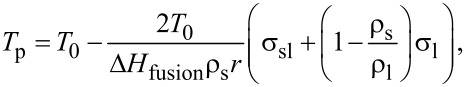


where *T*_0_ is the bulk melting temperature of a metal, Δ*H*_fusion_ is the latent heat of fusion, ρ_s_ and ρ_l_ are the densities of solid and liquid metal, respectively, σ_sl_ is the solid–liquid interfacial energy and σ_l_ is the surface energy of the liquid [50–51]. Figure 2 demonstrates the melting temperature of iron, nickel, gold and silver particles as a function of the diameter [50]. It is seen in Figure 2 that the melting temperature is decreased from the bulk value for particles with a diameter below 100 nm, and a noticeable decrease is observed below 10 nm. On the basis of this calculation, the authors of [50] conclude that the catalytic particles with diameter of 1–3 nm should be in a liquid form at typical synthesis conditions of nanotubes.

Secondly, the melting point of catalytic particles is decreased by forming a eutectic with carbon [52]. In [52], it was calculated that melting points of iron particles with diameters of 1–2 nm, which catalyze the SWCNT growth, can be reduced by up to 700–800 °C, down to 550 °C. This trend was confirmed by molecular dynamics simulations [53–55]. There are also experimental reports on the presence of catalytic particles in the liquid state during the growth of MWCNTs [27] and SWCNTs [56].

The authors of [52] suggest that for the bulk CVD such as in the injection methods for growing SWCNTs that use temperatures in the order of 1000 °C [57–58], the catalyst is likely to be in the liquid state. However, in situ TEM observations on the growth of SWCNTs and MWCNTs by the catalytic thermal decomposition of hydrocarbons on metallic and carbidic nanoparticles at temperatures up to 650 °C demonstrated that the particles remained crystalline during the growth process, although their shape was modified [41,59–62]. In particular, it was shown that crystalline Ni nanoparticles with a size down to ≈4–5 nm catalyzed the growth of nanotubes at temperatures as high as 540 °C [41] and 615 °C [59]. The authors of [61] observed the growth of SWCNTs with a diameter as small as 1.5 nm on the solid Fe_3_C nanoparticle that exhibited structural fluctuations at 600 °C. Also, they observed the growth of ≈15–20 nm diameter MWCNTs on the crystalline Fe_3_C nanoparticles. The TEM data testified that carbon atoms migrated through the bulk of nanoparticles during the nanotube growth.

**Figure 3:**
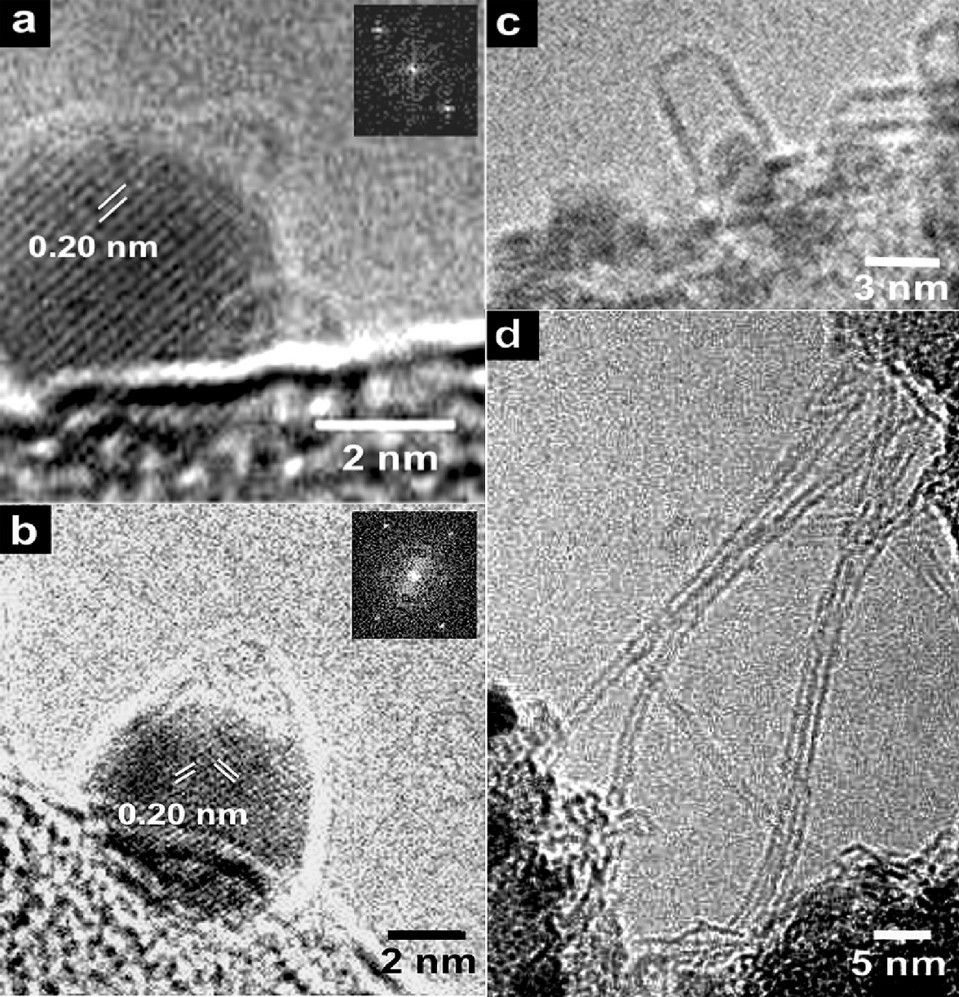
(a,b) Environmental TEM images of Ni crystalline nanoparticles recorded at 615 °C. White lines mark crystalline lattice fringes and numbers denote spacing between neighboring fringes. The insets present FFTs of the corresponding particles. (c,d) Ex situ HRTEM micrographs obtained for the same sample. Reprinted with permission from [59], copyright 2007 American Chemical Society.

Figure 3 presents environmental and high-resolution TEM images of various stages of SWNT growth on Ni catalytic particles [59]. The ETEM images in Figure 3a,b recorded at 615 °C show Ni particles for which SWCNT nucleation has stopped early. On top of each catalyst particle, a small-sized carbon cap is visible. Crystalline lattice fringe contrast is seen in the Ni particle, as marked by white lines. The authors of [59] assigned strong reflections in the fast Fourier transform (FFT) of Figure 3b to {111} planes, with the face-centered cubic (fcc) Ni lattice oriented close to the [110] axis. Figure 3c,d show ex situ HRTEM images of SWCNTs. Figure 3c presents an individual hemispherically capped SWCNT at a more progressed stage of growth. It is oriented tangentially to the Ni catalyst cluster. Figure 3d demonstrates low-magnification image of several synthesized nanotubes.

**Chemical state of catalyst.** The chemical state of catalyst during the nanotube growth was actively debated. The following three main questions were discussed. (i) Whether metallic catalyst particles do transform to carbide particles during the growth process? (ii) Whether sub-surface intermediate carbide is formed on the metallic particles? (iii) Whether the synthesis on purely metal carbide catalytic particles is possible?

**Figure 4:**
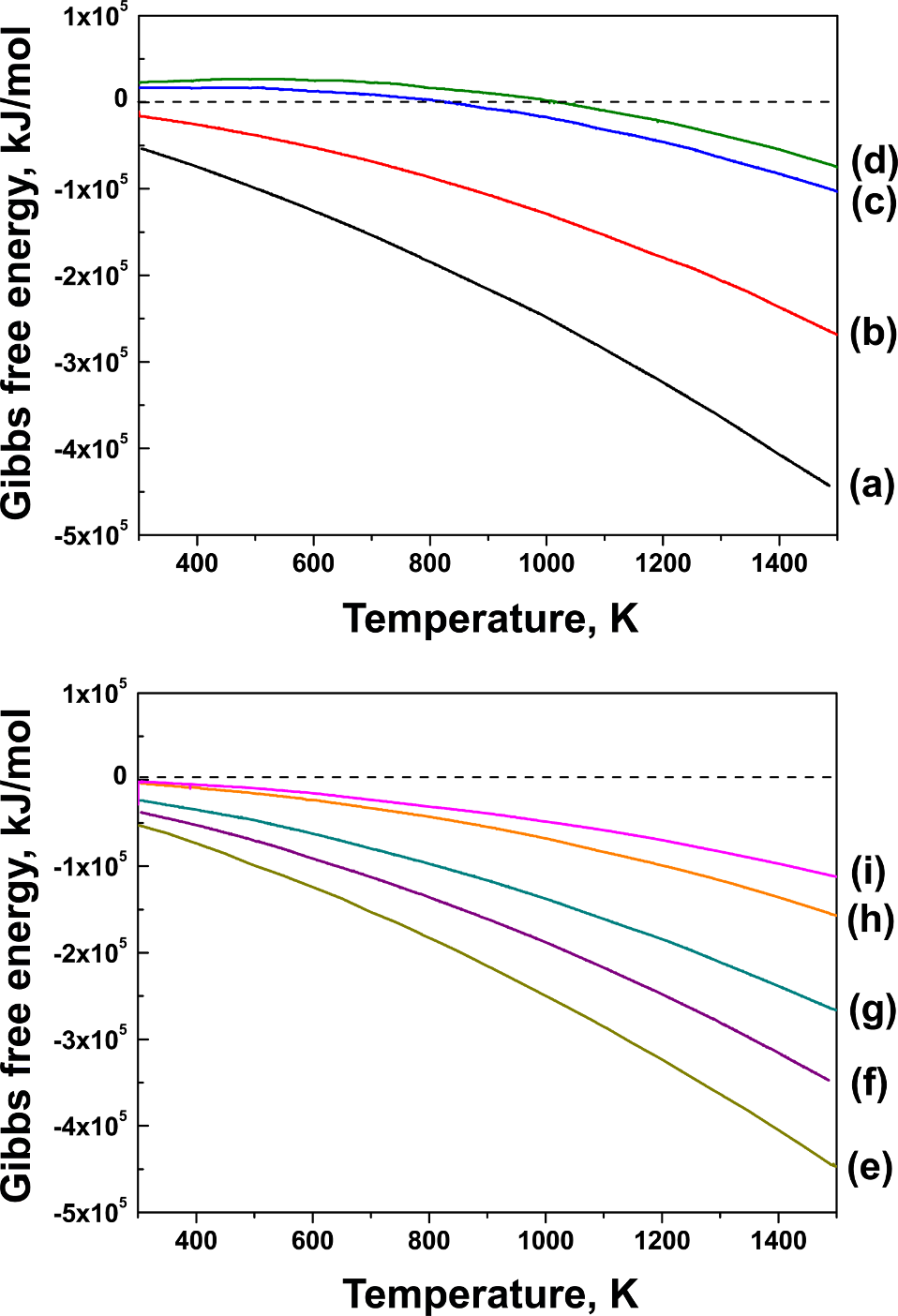
Calculated changes in Gibbs free energy for the reaction of Ni with (a) C_2_H_2_, (b) C_2_H_4_, (c) CH_4_ and (d) CO. Calculated changes in Gibbs free energy for the reaction of C_2_H_2_ with (e) Ni, (f) Co, (g) Fe, (h) W and (i) Mo. The data are replotted from [65].

Despite the fact that several authors reported that purely metallic particles catalyze the nanotube growth [52,59,63–64], the authors of [65] performed X-ray diffraction studies (XRD) of catalytic nanoparticles of different chemical elements and showed that “typical” catalysts such as Fe, Ni and Co underwent carburization during the induction phase of the synthesis (the period until the achievement of carbon precipitation), which disappeared after the growth process. In all cases, the metal underwent carburization before the growth of nanotubes was initiated. However, the authors of [65] mentioned that the core of nanoparticles possibly remained as pure metal. For “atypical” catalysts such as W, the carburization was observed both after induction and growth of nanotubes. On the basis of standard thermodynamic data, the authors of [65] concluded that any purely metallic catalyst should become carburized under common growth temperatures of nanotubes. Figure 4a–d shows the changes in Gibbs free energy for the reaction between Ni and different carbon precursors (CO, CH_4_, C_2_H_4_ and C_2_H_2_). According to these data, nickel carbide forms under a broad range of temperatures for the reaction with C_2_H_4_ and C_2_H_2_ (Figure 4a,b), while temperatures higher than 800 K are needed for the reaction with CO and CH_4_ (Figure 4c,d). The negative changes in Gibbs free energy increase in the line with CO, CH_4_, C_2_H_4_ and C_2_H_2_. This explains why C_2_H_2_ is one of the most reactive carbon precursors for the nanotube synthesis. The changes in Gibbs free energy for the reaction between C_2_H_2_ and different metallic catalysts (Ni, Co, Fe, W and Mo) are presented in Figure 4e–i. The formation of metal carbides is predicted at the elevated temperatures during nanotube growth for all these metals. The largest increases in Gibbs free energy are predicted for the reactions with Ni, Co and Fe [65].

The transformation of purely metallic catalysts into metal carbides with their subsequent decomposition before the nanotube growth was also observed by other authors [66–69]. This implies the decomposition of metal carbides as an elementary step of carbon nanotube synthesis [69]. In [66–67], XRD studies revealed the formation of iron oxides and carbide before the nanotube growth. Iron carbide was observed immediately before the start of the growth [67], and the process of its decomposition to Fe and graphite coincided with the onset of the nanotube growth [66]. In [68], time-resolved XPS studies showed the formation of chemisorbed carbon on Fe catalyst and carbidic carbon before the nanotube growth, with their further transformation to sp^2^ graphitic carbon network.

The question of the formation of the intermediate metastable carbidic phases during the nanotube growth was also actively debated. In the last decades, it was reported that metal nanoparticles can undergo partial carburization, i.e., the chemical transformation of metal into metal carbide, and subsequent reverse decomposition during the synthesis of carbon filaments and nanotubes by the catalytic thermal decomposition of hydrocarbons. In 1970–1980s, the growth of carbon filaments on metallic iron catalyst was actively studied by Buyanov and Chesnokov, and a carbide cycle mechanism of the growth was proposed [70–75]. According to this mechanism, a metastable carbide-like intermediate compound was formed in the subsurface layer of a catalytic particle as a result of the decomposition of hydrocarbon. The decomposition of intermediate carbide led to the supersaturation of metal by carbon. Indeed, the degradation of iron carbide Fe_3_C results in the mixture of carbon and iron with a carbon content of 6–7 wt %, whereas the saturated solid solution of carbon in iron contains not less than 0.025 wt % carbon [75]. Thus, a large carbon concentration gradient was created in the bulk of the catalytic particle. This caused the diffusion of carbon atoms from the surface where the hydrocarbon was decomposed through the bulk of metal to the sites of the crystallization into a graphite phase (carbon filaments). The degraded intermediate carbide was restored as a result of the decomposition of hydrocarbon, and this cyclic process took place as long as there were the gaseous source of carbon and active catalyst in the system. A carbide cycle mechanism was proven for the growth of carbon filaments by the decomposition of different hydrocarbons (methane, butane, propylene, isobutylene, butadiene, benzene) on iron catalyst [75]. Other authors also reported the formation of intermediate iron carbide phases during the growth of filaments [76–80]. More recent studies on the CVD growth of nanotubes evidenced the presence of intermediate iron carbide and discussed its role in the tube formation [65,69,81–84].

A smaller number of reports was dedicated to the investigation of the chemical state of nickel catalyst during the growth of carbon filaments and nanotubes. The formation of intermediate carbide phases was revealed for nickel catalysts, as in the aforementioned cases of iron catalysts. Buyanov and Chesnokov reported that the above-described carbide cycle mechanism is applied for the growth of carbon filaments on metallic nickel catalyst [75,85–87]. The authors of [88] also observed the formation of intermediate nickel carbide during the growth of filaments. Recent studies on the growth of nanotubes by the CVD method confirmed the presence of an intermediate nickel carbide phase in the nickel catalyst [65,69,84].

The presence of intermediate carbide phases was also reported for cobalt catalysts. Buyanov and Chesnokov suggested that the carbide cycle mechanism could be applied to the growth of carbon filaments on all iron-group metal catalysts, including cobalt [75]. The authors of recent studies on the growth of nanotubes by the CVD method [65,69,84] also showed that iron, cobalt and nickel catalysts followed a similar reaction path with the formation of intermediate carbide phases during the synthesis process.

It should be noted that the formation of intermediate carbide phases was not usually confirmed by in situ TEM analysis of the nanotube growth on nickel catalyst [41,59,64]. This is probably caused by the fact that metal and carbide have rather similar lattice constants and thus they can not be easily distinguished by diffraction and TEM [52], especially in the case of partial carburization of catalyst particle at its surface [65]. However, in situ TEM confirmed the structure of iron and cobalt carbides when they were the active catalyst phase of the nanotube growth [61–62,81,89–91].

Some authors reported that iron carbide formed from metallic iron did not decompose and thus it was not an intermediate phase, but served as catalyst of the nanotube growth [67,89–90,92]. The stability of Fe_3_C structure was explained by special synthesis conditions, in particular high pressure of hydrocarbon and too low synthesis temperatures for the decomposition of iron carbide [89], which is known to be stable until ≈700–750 °C [93–94]. In [90], it was demonstrated that the growth mechanism of nanotubes depended on the phase composition of iron catalyst nanoparticles. It was found that for γ-Fe rich mixtures, metallic iron was the active catalyst phase for the tube growth, implying that the transformation to iron carbide was not necessary (however, the formation of subsurface carbon-rich phases and bulk Fe-C solid solutions were not excluded). In contrast, for α-Fe rich mixtures, Fe_3_C formation was dominant and constituted the part of the growth process. On the basis of the data, it was concluded that kinetic effects dominated the catalyst phase evolution.

**Figure 5:**
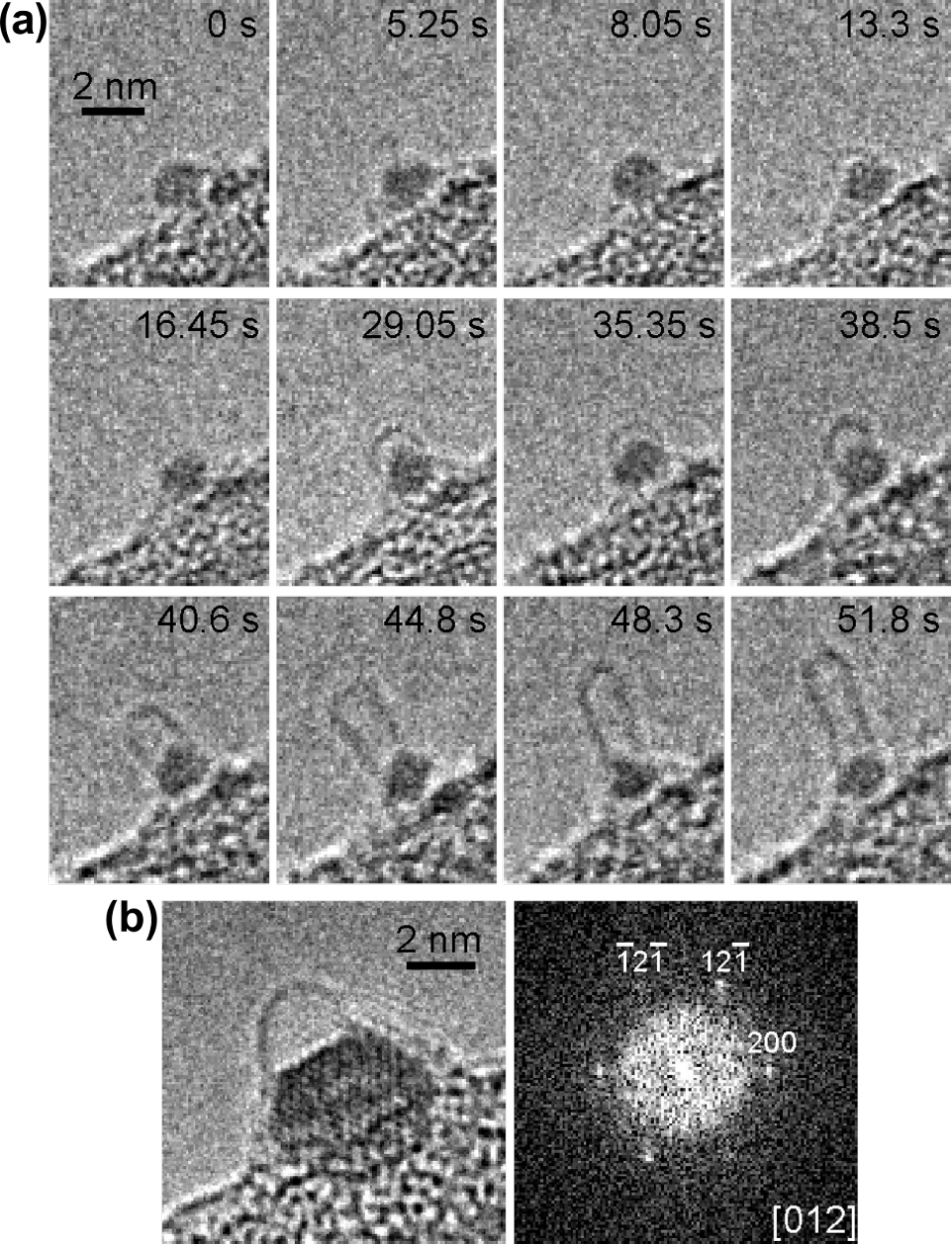
(a) In situ HRTEM micrographs of the nucleation and growth of an individual SWCNT on the catalyst nanoparticle. The recording time of snapshots is denoted. (b) A micrograph of the nanoparticle with a carbon dome. The particle exhibits the lattice image and the respective extra diffraction in the Fourier transform. The particle is identified as iron carbide Fe_3_C viewed along the [012] direction. Reprinted with permission from [61], copyright 2008 American Chemical Society.

Other authors also proved that metal carbide can be an active catalyst for the nanotube growth [61–62]. The authors of [61] performed the synthesis of SWCNTs and MWCNTs using C_2_H_2_ as carbon source and iron carbide catalyst. Figure 5a shows in situ HRTEM micrographs of the growth process of individual SWCNT. Before the nucleation of SWCNT, the catalyst nanoparticle shows in every snapshot different facets (e.g., *t* = 8.05 and 16.45 s). Various carbon cages jut out from the particle frequently and disappear in a few seconds (*t* = 5.25, 13.3 and 29.05 s). The unstable carbon cage and particle change their shape rapidly (e.g., *t* =13.3 and 29.05 s). After an incubation period, the stable dome, which is the nucleus of SWCNT, appears at *t* = 35.35 s. It grows gradually into 1.5 nm diameter SWCNT with a length of 3.6 nm (from *t* = 40.6 s to *t* = 51.8 s) [61].

Figure 5b demonstrates a micrograph of the nanoparticle with a carbon dome. The particle exhibits the lattice image and the respective extra diffraction in the Fourier transform. The particle is identified as iron carbide Fe_3_C viewed along the [012] direction [61].

**Tip- and base-growth models.** Two growth models were reported for the formation of nanotubes on catalysts with a substrate, which differ in the position of growing nanotube relative to the catalytic particle: tip- and base-growth models [34,45]. In the tip-growth model, precursor molecules dissociate at the active face of catalyst particle. The carbon is dissolved, diffuses through the bulk and is incorporated into a growing nanotube. This mechanism pushes the catalyst particle that resides at the growing tip further away from the substrate. The growth of the nanotube continues as long as fresh feedstock is supplied, unless the catalyst particle becomes deactivated by an impermeable carbon shell. In the base-growth model, the initial precursor dissociation and carbon diffusion occur similarly to those in the tip-growth model, but the carbon precipitation and nanotube formation do not lead to lifting the catalytic particle from the substrate. Carbon precipitates on the apex of the metal, as far as possible from the substrate. The nanotube growth starts from the formation of a hemispherical dome, which is the most preferable closed-carbon structure on a spherical particle. Subsequent hydrocarbon dissociation occurs on the lower surface of the particle, and carbon atoms diffuse upward in the metal. This leads to the elongation of the nanotubes above the particle that remains attached to the substrate [34].

The interaction between the catalyst particle and substrate decides whether the nanotube growth will follow the tip- or base-growth mechanism [34,45]. When the interaction is weak (there is an acute contact angle between the catalytic particle and substrate), the tip-growth model is realized. When the interaction is strong (there is an obtuse contact angle between the particle and substrate), the base-growth model is favored [34]. The growth of MWCNTs from Fe catalyst was observed to follow either growth mechanism on different substrates. Namely, the tip-growth on SiO_2_ and base-growth on Ta [95]. The authors of [95] found that the catalytic particles on Ta had a hemispherical shape, whereas the particles on SiO_2_ had a bead shape. The contact angles of the Fe catalyst particles with the SiO_2_ and Ta substrates revealed that the tip-growth was observed when the surface energy of the bare substrate was smaller than that of the catalyst-substrate interface and the base-growth was observed in the opposite case.

**Figure 6:**
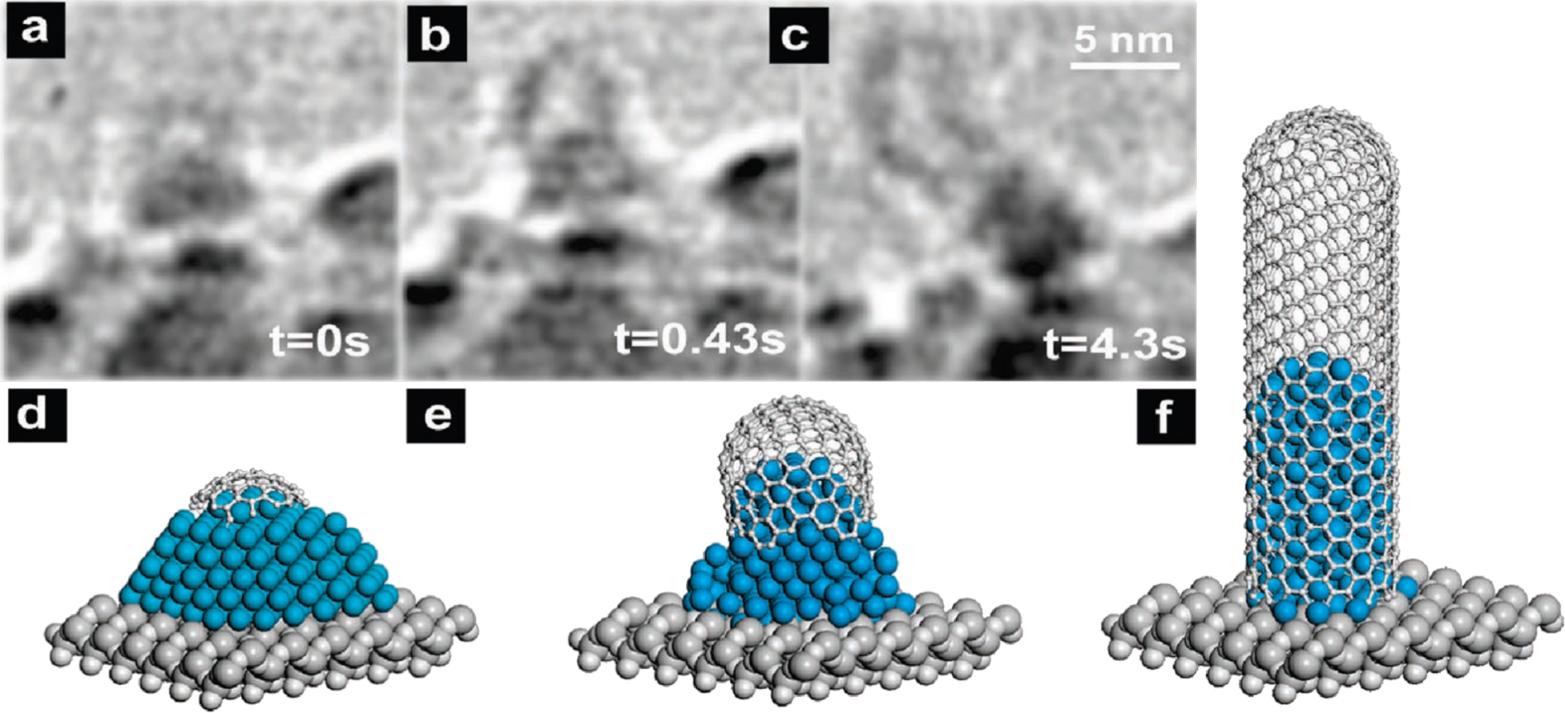
(a–c) The environmental HRTEM image sequence of the consecutive stages of the base-growth of SWCNT on Ni catalytic nanoparticle with SiO_x_ substrate using C_2_H_2_ as carbon precursor at 615 °C. The sequence was extracted from a continuous video recording. The time of the corresponding snapshots is denoted. (d–f) The schematic ball-and-stick model of the stages of the nanotube growth. Reprinted with permission from [59], copyright 2007 American Chemical Society.

The base-growth was reported for MWCNTs in [61,96–97] and SWCNTs in [29,59,61,64,98]. The tip-growth was observed for MWCNTs in [41,59–60,81] and SWCNTs in [99–101]. In [41,59–61,64,99], time-resolved in situ HRTEM was employed for the investigation of the nanotube growth and was shown to be a powerful technique for revealing the growth mechanism. The authors of [59] used environmental HRTEM to study the base-growth of SWCNTs from the acetylene decomposition on Ni nanoparticles with SiO_x_ substrate at 615 °C. Figure 6 demonstrates the HRTEM image sequence of the consecutive stages of the growth, which was extracted from a continuous video recording [59]. The SWCNT growth started from the formation of a carbon cap on the apex of the triangular/pyramidal metallic particle. It replicated the shape of the apex and had smaller diameter than the particle (Figure 6a). The apex of the particle acquired a cylindrical shape, lifting the carbon cap from the particle and forming the nanotube. The growing SWCNT forced further cylindrical reshaping of the particle, which led to increasing the contact angle of the particle with the substrate to approximately 90° (Figure 6b). The growth process stopped when the nanotube encapsulated the particle down to its substrate interface (Figure 6c). The schematic representation of the above-described stages of the SWCNT growth in a ball-and-stick model is shown in Figure 6d–f [59].

It should be noted that although the base-growth of SWCNTs was commonly observed in a number of reports [29,59,61,64,98], the authors of [99–101] demonstrated the growth of SWCNTs by the tip-growth mechanism. In [101], long and aligned SWCNTs were synthesized by the fast-heating CVD process. It was proven that although both growth mechanisms coexisted in the experiments, long and oriented nanotubes were produced only by the tip-growth mechanism. In [100], SWCNTs were synthesized by the low-temperature CVD process using two different types of Co_x_Mg_1−x_O catalysts prepared by atomic layer deposition and impregnation. It was shown that the catalyst prepared by atomic layer deposition catalyzed the growth of SWCNTs by the tip-growth mode, whereas the catalyst prepared by impregnation catalyzed the base-growth of nanotubes. This was explained by weak interactions between Co nanoparticles and MgO support in the catalyst prepared by atomic layer deposition and extremely strong metal-support interactions between epitaxial Co nanoparticles and MgO support in the catalyst prepared by impregnation.

**Tangential and perpendicular growth modes.** In the recent years, the other two growth modes relying on the diameter ratio between SWCNT and catalyst particle size has become more and more important in controlling SWCNT diameter and even chirality [102–104].

In [102], the statistical analysis of the TEM data was conducted in order to elucidate the correlation between the sizes of SWCNTs or nuclei and the nanoparticles on which they grow. They proved the existence of two nucleation and growth modes of nanotubes: tangential and perpendicular modes. In the tangential growth mode, the carbon wall of growing nanotube is oriented tangentially to the surface of nanoparticle. As a result, the diameter of grown nanotube is close to that of the nanoparticle. In the perpendicular growth mode, the carbon wall of growing nanotube is oriented perpendicular to the surface of nanoparticle. As a result, the diameter of grown nanotubes is smaller or not correlated with that of the nanoparticle. From statistical observations it was concluded that the growth mode is perpendicular if the ratio of the diameters of nucleus of the nanotube and nanoparticle is lower than 0.75. It was shown that both growth modes do not depend on the diameter of nanoparticle. The growth mode was demonstrated to be dependent on the synthesis time. At short times (40 s and 2 min), the perpendicular growth was observed, whereas at long times (10 and 30 min) the tangential mode was dominant. Using tight binding Monte Carlo simulations, it was shown that the tangential growth occurs at reaction conditions that are close to equilibrium, whereas the perpendicular growth occurs at conditions driven by kinetic effects. On the basis of the data, the authors of [102] concluded that the control of chirality of nanotubes should be searched at reaction conditions that are close to thermodynamic equilibrium, when the tangential growth is favored.

The authors of [103], investigated the correlation between the growth mode and the lengths of SWCNTs. Using TEM, they showed that the length of SWCNTs depended on the ratio of diameters of nanotube and nanoparticle, i.e. the growth mode. The SWCNTs grown in perpendicular mode were much longer than those grown in tangential mode. Using Monte Carlo computer simulations, the authors of [103] demonstrated that nanoparticles with low carbon concentration (4%) catalyzed the tangential growth of SWCNTs, where the particle wets the inner wall of nanotube and can be easily passivated by encapsulating graphitic layers, which leads to stopping of the growth and formation of short SWCNTs. In contrast, the nanoparticles with high carbon concentration (18%) catalyzed the perpendicular growth of SWCNTs and kept their activity for longer time, which led to the formation of long SWCNTs.

In [104], the control of the growth mode of SWCNTs led to the synthesis of semiconducting SWCNTs with a narrow band-gap distribution. SWCNTs were grown on acorn-like partially carbon-coated Co nanoparticles. The inner Co particle was an active catalytic phase, whereas the outer carbon layer prevented the aggregation of particles and ensured a perpendicular growth mode. As a result, the grown SWCNTs had a very narrow diameter distribution centered at 1.7 nm and high content of semiconducting fraction of >95%. The range of band gaps of SWCNTs was <0.08 eV. They demonstrated an excellent thin-film transistor performance.

**Chirality selective growth.** The synthesis of SWCNTs with specific chiralities is currently a very active research field. This section reviews the reports on the chirality selective growth of SWCNTs and discusses the growth mechanism of nanotubes.

In 2003, Bachilo and co-authors synthesized SWCNT samples with a great abundance of the (6,5) and (7,5) nanotubes on CoMo catalyst [105]. Since then, chirality selective growth of SWCNTs was succeeded on a number of different catalysts: CoMo [106–108], FeCo [109], FeRu [110], NiFe [111], Co [112–114], FeCu [115], Au [116], CoMn [117], Ni [118], Fe [119–120], CoPt [121], Co_x_Mg_1−x_O [122], CoSO_4_ [123], WCo alloy [124–125] and Mo_2_C [126]. SiO_2_ or MgO were used as catalyst support. The synthesis was conducted using different carbon precursors: CO [105–108,112–115,117–119,122–123], C_2_H_5_OH [107,109,120–121,124–126], CH_3_OH [107], CH_4_ [110,116] and C_2_H_2_ [111]. Table 1 summarizes the reports on the chirality selective synthesis of SWCNTs.

**Table 1:** Summary of reports on chirality selective synthesis of SWCNTs by the CVD method. Given are the carbon feedstock, catalyst, catalyst support, synthesis temperature and main chirality of synthesized nanotubes in a chronological order.

Carbon feedstock	Catalyst	Catalyst support	Synthesis temperature	Main nanotube chirality^a^	Ref.

CO	CoMo	SiO_2_	750 °C	(6,5)*, (7,5)	[105]
C_2_H_5_OH	FeCo	USY-zeolite	650 °C	(6,5)*, (7,5)	[109]
			750 °C	(6,5), (7,5)*, (7,6)	
			850 °C	(7,5)*, (7,6), (8,6), (8,4), (9,4)	
CO	CoMo	SiO_2_	700 °C	(6,5)*, (6,6), (7,7)	[106]
			750 °C	(6,5)*, (8,4), (6,6), (7,7)	
			800 °C	(6,5)*, (6,6), (7,7)	
			850 °C	(7,5), (7,6)*, (8,6), (8,7), (6,6), (7,7)	
		MgO	750 °C	(6,5), (7,5)*, (6,6)	
CO	CoMo	SiO_2_	800 °C	(7,5), (7,6)*, (8,4)	[107]
C_2_H_5_OH					
CH_3_OH					
CH_4_	FeRu	SiO_2_	600 °C	(6,5)*	[110]
			700 °C	(6,5)*, (7,5), (8,4)	
			850 °C	(7,5)*, (7,6), (8,4)	
CO	CoMo	SiO_2_	800 °C	(6,5)*, (7,5), (7,6)	[108]
C_2_H_2_	NiFe		600 °C	(7,5)*, (8,4), (7,6), (8,3), (6,5) (Ni_0.5_Fe_0.5_)	[111]
				(8,4)*, (7,5), (6,5), (7,6), (8,3) (Ni_0.27_Fe_0.73_)	
CO	Co	MCM-41 (mesoporous SiO_2_)	550 °C	(6,5)*, (8,4)	[112]
			650 °C	(6,5)*, (7,5), (8,4)	
			750 °C	(6,5), (7,5)*, (7,6), (8,4), (8,6)	
			850 °C	(7,5), (7,6)*, (8,4), (8,6)	
			950 °C	(7,5), (7,6)*, (8,4), (8,6)	
CO	FeCu	MgO	600 °C	(6,5)*	[115]
			750 °C	(6,5), (7,5)*, (7,6), (8,3), (8,4)	
			800 °C	(6,5), (7,5)*, (7,6), (8,3), (8,4), (8,6), (9,4)	
CO	Co	TUD-1 (mesoporous SiO_2_)	800 °C	(9,8)*	[113]
CH_4_	Au	SiO_2_	700–750 °C	(6,5)*	[116]
CO	CoMn	MCM-41 (mesoporous SiO_2_)	600 °C	(6,5)*, (7,3), (8,3)	[117]
			700 °C	(6,5)*, (7,3), (8,3)	
			800 °C	(6,5)*, (7,5)	
CO	Ni	SiO_2_	500 °C	(6,5)*, (7,5)	[118]
CO	Fe		880 °C	(13,12)*, (12,11), (13,11)	[119]
CO	Co	SiO_2_	600 °C	(6,5)*, (7,5), (6,4), (7,6), (8,3), (8,4)	[114]
C_2_H_5_OH	CoPt	SiO_2_	800 °C	(6,5)*, (7,5), (7,6)	[121]
			850 °C	(6,5), (7,5), (7,6)*	
CO	Co_x_Mg_1−x_O		400 °C 500 °C 600 °C	(7,6)*, (9,4) (6,5)* (6,5)*, (7,5), (8,3)	[122]
CO	CoSO_4_	SiO_2_	780 °C	(9,8)*	[123]
C_2_H_5_OH	WCo alloy	SiO_2_	1030 °C	(12,6)*	[124]
C_2_H_5_OH	WCo alloy	SiO_2_	1050 °C	(16,0)*	[125]
C_2_H_5_OH	Mo_2_C	SiO_2_	850 °C	(14,4), (13,6), (10,9)	[126]
C_2_H_5_OH	Fe	SiO_2_	850 °C	(15,2)*	[120]

^a^Asterisk marks the dominant nanotube chirality.

In early and many later works the synthesis of samples of near-armchair SWCNTs with predominant (6,5) chirality was reported [105–110,112,114–118,121–122]. The mechanism of preferential growth of near-armchair SWCNTs is still debated. In [109], the effect was explained by the stability of cap structures of near-armchair nanotubes, which are formed on the catalyst before the growth of the tube wall, as compared to near-zigzag tubes and a small number of possible cap structures for small diameter tubes. Theoretically, the authors of [127] showed that some caps are preferentially stabilized due to their epitaxial relationship to the solid catalyst surface, and the growth of corresponding tubes is favored. In [128], on the basis of the dislocation growth mechanism, it was shown that the abundance of near-armchair nanotubes in the synthesized samples is caused by their higher growth rates as compared to near-zigzag tubes. This trend was proven by several experimental studies [129–130]. Therefore, the chiral selectivity can be related to the nucleation of carbon species on catalytic particles and the different growth rates depending on the chiral angle of nanotubes. In other words, the chirality selective growth of SWCNTs is realized through either thermodynamic control, such as building a more stable tube-catalyst interface or kinetic control, such as different growth rates of different SWCNTs. Recently, the authors of [131] combined thermodynamic (preference to low energy) and kinetic (preference to higher rate) arguments within a unified theoretical model, which explains the preferential growth of near-armchair nanotubes.

It was shown that chirality selectivity is influenced by the synthesis parameters: gaseous carbon source [106–107], its pressure [108], catalyst composition [111], type of support [106] and synthesis temperature [106,109–110,112,115,117,121–122]. The authors of [107] synthesized SWCNTs using four different carbon precursors: CO, C_2_H_5_OH, CH_3_OH and C_2_H_2_ on CoMo catalyst. Narrowly (*n*,*m*) distributed SWCNTs were obtained only using CO, C_2_H_5_OH and CH_3_OH. In samples synthesized using CO the (7,6), (7,5) and (8,4) tubes dominated, whereas the samples obtained using C_2_H_5_OH and CH_3_OH contained more (8,6), (9,5) and (8,7) nanotubes. In [106], it was shown that the CH_4_ feed did not result in such a narrow (*n*,*m*) distribution dominated by near-armchair nanotubes as the CO feed.

The authors of [108] performed a systematic study of the chirality distribution of SWCNTs varying the pressure of CO feed on CoMo catalysts between 2 and 18 bar. Three nanotube chiralities (6,5), (7,5) and (7,6) were dominant in the samples. However, their relative content depended on the pressure of the carbon feedstock. The (6,5) tube had the largest content at 18 bar CO and its content decreased with decreasing the pressure from 18 to 2 bar. In contrast, the (7,6) tube had the largest content at 2 bar CO and its content decreased with the increase of CO pressure. The yield of the (7,5) tube was the largest at 12 bar CO pressure.

The authors of [111] investigated the changes in the chirality distribution of SWCNTs by tuning the composition of Ni_x_Fe_1−x_ catalytic nanoparticles. They showed that pure Ni catalyst yielded a relatively wide chirality distribution of SWCNTs, where the (9,4) tubes dominated and smaller amounts of (8,4), (7,5), (10,2), (8,6), (9,5) and (10,3) tubes were present. The Ni_0.67_Fe_0.33_ catalysed sample showed a similar chirality distribution with dominating (7,6) tubes. In comparison, the samples obtained with Ni_0.5_Fe_0.5_ and Ni_0.27_Fe_0.73_ catalysts were characterized by dramatic changes in chirality distributions. The sample obtained with Ni_0.5_Fe_0.5_ was composed of mainly (7,5) and (8,4) tubes with smaller amounts of the (7,6), (8,3) and (6,5) tubes. The sample grown with Ni_0.27_Fe_0.73_ has a much narrower chirality distribution with dominating (8,4) tube and smaller amounts of (7,5), (6,5), (7,6) and (8,3) tubes. The authors of [111] suggested that changes in the catalyst structure, which are a result of the tuning of the catalyst composition, affected the lattice mismatch of the catalyst with certain nanotube chiralities and led to the observed changes in the chirality distribution.

In [106], the effect of catalyst supports, such as SiO_2_ and MgO, on the chirality distribution of SWCNTs was studied. The difference in the morphology of these catalyst supports resulted in the growth of different SWCNTs. In both cases, SWCNTs with near-armchair chiralities were obtained. In the samples synthesized with SiO_2_ support, the (6,5) tubes dominated. The samples obtained using MgO support contained less (6,5) tubes and more (7,5), (8,4) and (6,6) tubes. The average diameters of these four nanotubes are similar, but the chiral angle was reduced in the MgO sample.

In most works, it was observed that the increase in the synthesis temperature led to increase in the nanotube diameters and broadening of the chirality distribution [106,109–110,112,115,117,121–122]. The (6,5) nanotube dominated in the samples synthesized at temperatures around 500–700 °C, whereas such selectivity disappeared at higher temperatures. In [121], a bimetallic CoPt catalyst was suggested for the selective growth of the (6,5) tubes at synthesis temperatures as high as 800–850 °C. The formation of CoPt alloy and its improved stability was suggested to be responsible for the selective growth of small diameter SWCNTs with a narrow chirality distribution.

The authors of recent works [113,119–120,123–126] succeeded in the selective growth of SWCNTs with chiralities that are different from (6,5). In [113,123], near-armchair SWCNTs with a chirality of (9,8) were selectively synthesized. The authors of [113] produced the SWCNT samples using Co catalyst on TUD-1 (mesoporous SiO_2_) support. 59.1% of semiconducting SWCNTs had the (9,8) chirality. It was suggested that strong metal-support interaction stabilized the Co clusters with a narrow diameter distribution around 1.2 nm, which were responsible for the selective growth of the (9,8) tubes. In [123], the (9,8) nanotubes were selectively synthesized on CoSO_4_ catalyst supported by SiO_2_ with 51.7 % abundance among semiconducting SWCNTs. The chirality selectivity was explained by the formation of Co particles with an average size of 1.23 nm, which matched the diameter of the (9,8) tube. Additionally, the presence of sulfur, which limited the aggregation of Co particles and formed Co–S compounds, was suggested to enable the chirality selectivity toward the (9,8) tubes.

In [119], large diameter SWCNTs with a narrow (*n*,*m*) distribution and dominant (13,12) tubes (*d* = 1.67 nm) were synthesized in aerosol floating-catalyst CVD process with a use of ferrocene as catalyst precursor and a small amount of ammonia. Over 90% of SWCNTs had near-armchair structure. It was suggested that NH_3_, which is a strong etchant, selectively etched off SWCNTs with small chiral angles due to their higher reactivity and lower stability as compared to high chiral angle tubes. The same applied to small diameter nanotubes due to their higher curvature. Additionally, the presence of NH_3_ could affect the catalyst clusters already during nucleation, suppressing the growth of tubes with small chiral angles.

In most recent works, efforts were aimed at the optimization of the SWCNT-catalyst interface for the chirality-selective growth. The authors of [124–125] used WCo alloy particles with specific structure as template to realize the chirality-controlled growth of SWCNTs. In [124], the (12,6) tubes (*d* = 1.28 nm) with an abundance higher than 92% were selectively synthesized using ethanol as carbon source. W_6_Co_7_ alloy nanoparticles were found to be responsible for catalyzing the nanotube growth. It was suggested that the selective growth was a result of good structural match between the arrangement of carbon atoms around the circumference of nanotube and the arrangement of metal atoms of the nanocrystal catalyst.

In contrast to most previous reports on the selective growth of near-armchair nanotubes, the authors of [125] synthesized zigzag nanotubes with chirality of (16,0) using W_6_Co_7_ catalyst. The abundance of the (16,0) tubes in the samples was estimated to be ≈80%. It was suggested that the (116) planes of the nanocrystal catalyst acted as templates for the (16,0) tubes due to the structural match between the open end of the tube and the arrangements of metal atoms of the (116) planes of the catalyst. The authors of [125] noted that the structural match between the tubes and nanocrystal catalyst represented the thermodynamic ascendancy for the growth of SWCNTs with specific chiralities, but the growth kinetic was also important. They concluded that zigzag SWCNTs can be dominantly produced by combining the structural template effect of nanocrystal catalyst and the optimization of growth kinetics. The authors of recent report [120] also succeeded in the CVD synthesis of near-zigzag SWCNTs with the dominant chirality of (15,2) using Fe catalyst.

It should be noted that besides the CVD method the single chirality SWCNTs can also be obtained by the “cloning growth” and organic synthesis, as described in detail in review [132].

#### Inner tube growth inside SWCNTs

While the coalescence mechanism is generally accepted for the formation of inner tubes from fullerene-filled SWCNTs [133–142], only a few works discussed the mechanism of the inner tube growth from SWCNTs filled with other molecules.

In [143], the authors grew inner tubes via the thermally-induced chemical transformation of ferrocene molecules inside the host SWCNTs. They mentioned that ferrocene molecules are decomposed upon annealing, and they act as catalyst source and provide carbon atoms for the inner tube growth at the same time. From the analysis of ex situ HRTEM data, it was concluded that iron carbide catalyzed the inner tube growth.

**Figure 7:**
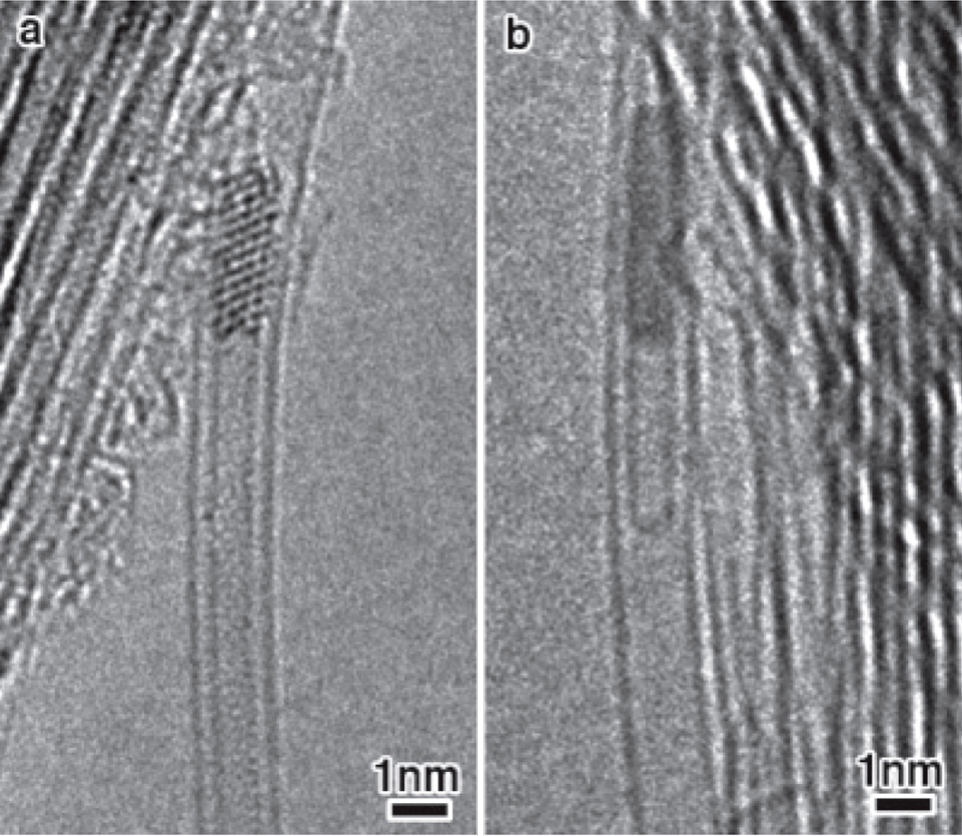
The HRTEM images of Pt acetylacetonate-filled SWCNTs ex situ annealed at 700 °C for 2 h (a) and in situ annealed at temperatures up to 760 °C (b). Reprinted with permission from [144], copyright 2010 Wiley-VCH Verlag GmbH & Co. KGaA, Weinheim.

The authors of [144] traced the growth process of inner tubes inside Pt acetylacetonate-filled SWCNTs by HRTEM. Figure 7a shows a room-temperature HRTEM micrograph of DWCNTs formed via the annealing of the filled SWCNTs at 700 °C. It is visible that the inner tube is connected with its open end to a nanocrystal inside a SWCNT. It was determined that the interplanar distances of the nanocrystal correspond to those of a Pt crystal. This proved that metallic Pt catalyzed the inner tube growth. In situ HRTEM further confirmed that the inner tube wall remained terminated at a Pt crystal even at the growth temperature of 760 °С (Figure 7b). The authors of [144] suggested that the growth of the inner tube stopped when the carbon source was depleted. They mentioned that this growth mechanism is different from conventional bulk-scale synthesis of SWCNTs. The key difference to conventional synthesis with uncontrolled catalyst particle is the exceptional stability of the growth mechanism. Without a templating outer nanotube, fluctuations in growth conditions result in a finite lifetime of the catalyst. And growth stops once the particle is deactivated by a passivating layer of carbon. However, inside the atomically tight tubular confinement provided the outer nanotube, the formation of a passivating carbon shell is sterically hindered. The templating provides prolonged catalyst lifetimes and the growth is maintained for many hours until all feedstock is consumed [144].

**Figure 8:**
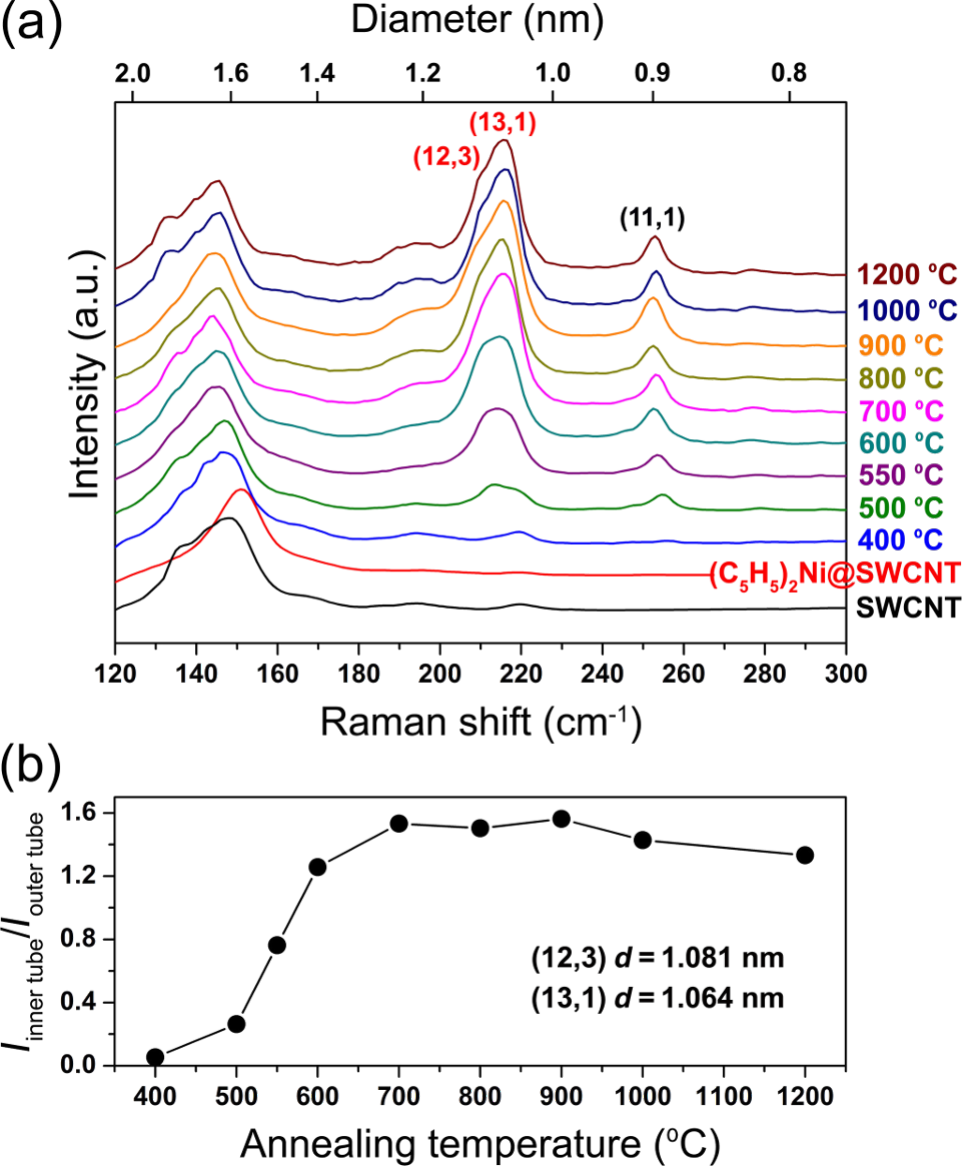
(a) The RBM-band of Raman spectra of the pristine, nickelocene-filled SWCNTs and samples annealed at temperatures ranging from 400 to 1200 °C for 2 h acquired at a laser wavelength of 633 nm. (b) The normalized area intensity of the RBM peak of the (12,3) and (13,1) inner tubes plotted versus annealing temperature. Reproduced from [145]. Published by The Royal Society of Chemistry under a Creative Commons Attribution 3.0 Unported License.

In [145], the nickelocene-filled SWCNTs were annealed at temperatures ranging from 250 to 1200 °C to form DWCNTs. Using Raman spectroscopy, it was shown that upon annealing the molecules reacted with one another and formed inner tubes inside the outer SWCNTs at a high yield. Figure 8a demonstrates the RBM-band of Raman spectra of the pristine, filled and annealed samples acquired at a laser wavelength of 633 nm (*E*_ex_ = 1.96 eV) [145]. The RBM-band of the pristine SWCNTs is positioned at frequencies between 125 and 160 cm^−1^. The RBM-band of the NiCp_2_-filled SWCNTs is shifted towards higher frequencies by 4 cm^−1^, which was previously reported for molecule-filled SWCNTs [143–144,146–147]. In the spectra of the annealed samples, additional peaks appear at 212, 216 and 253 cm^−1^, which correspond to inner tubes. The peak at 212 cm^−1^ was assigned to the (12,3) tube with a diameter of 1.08 nm, the peak at 216 cm^−1^ was attributed to the (13,1) tube with a diameter of 1.06 nm, and the peak at 253 cm^−1^ was assigned to the (11,1) tube with a diameter of 0.91 nm [145]. The diameter of the (12,3) and (13,1) tubes was close to the mean diameter, which allowed evaluating the formation of the major part of inner nanotubes. Figure 8b presents the relative area intensity of the RBM peak of the (12,3) and (13,1) tubes plotted versus annealing temperature [145]. The inner tubes grow fast with increasing temperature from 400 to 700 °C [145].

**Figure 9:**
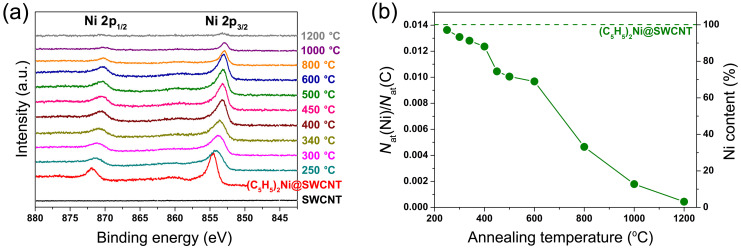
(a) The Ni 2p XPS spectra of the pristine, nickelocene-filled SWCNTs and samples annealed at temperatures between 250 and 1200 °C for 2 h. (b) The nickel-to-carbon atomic ratio *N*_at_(Ni)/*N*_at_(C) and nickel content plotted versus annealing temperature. The dashed horizontal line denotes the value for the NiCp_2_-filled SWCNTs. Reproduced from [145]. Published by The Royal Society of Chemistry under a Creative Commons Attribution 3.0 Unported License.

The evolution of the chemical state of the encapsulated compounds at every annealing step was analyzed by X-ray photoelectron spectroscopy. Figure 9a presents the Ni 2p spectra of the NiCp_2_-filled SWCNTs and samples annealed at temperatures between 250 and 1200 °C for 2 h [145]. The spectrum of the NiCp_2_-filled SWCNTs includes two peaks positioned at binding energies of 854.53 and 871.80 eV, which belong to the Ni 2p_3/2_ and Ni 2p_1/2_ edges, respectively. The Ni 2p spectra of the samples annealed at 250–340 °C demonstrate a successive downshift by up to 0.96 eV and broadening of the Ni 2p_3/2_ and Ni 2p_1/2_ peaks with increasing temperature. These features were explained by changes in the chemical state of nickel, because of the decomposition of NiCp_2_ with the formation of nickel carbides (Ni_x_C) [145]. In the spectra of the samples annealed at temperatures above 400 °C, the peaks are further downshifted and narrowed, and at 600 °C they reach the position of metallic nickel (Ni 2p_3/2_ peak is centered at ≈853 eV [148–149]). These observations were assigned to the chemical transformation of nickel carbides into metallic nickel [145], which was in agreement with previous reports that nickel carbides (in particular, Ni_3_C) are metastable [150] and that Ni_3_C degrades at temperatures above 400–500 °C [151–153]. At temperatures above 800 °C, nickel atoms are observed to be removed rather rapidly from the tubes, which is seen as decreased nickel Ni 2p signals to 3% of the initial value at 1200 °C. Figure 9b demonstrates the calculated nickel-to-carbon atomic ratio *N*_at_(Ni)/*N*_at_(C) and Ni content plotted versus annealing temperature [145]. For the NiCp_2_-filled SWCNTs, the nickel-to-carbon ratio amounts to 0.0141. At temperatures below 400 °C, the Ni content is decreased only to 90% or higher. At 450–600 °C, it is reduced to 69%. At higher temperatures, the loss becomes substantial. The Ni content drops to 33% at 800 °C, 13% at 1000 °C, and then at 1200 °C almost all nickel is released from the sample [145].

The study of the growth process of inner tubes inside the host SWCNTs facilitates understanding the growth mechanism of nanotubes. SWCNTs filled with organometallic molecules represent a unique system for the investigation of the growth mechanism of nanotubes. They are a stable system where the inner tube growth takes place with a slow enough rate over a long time. The synthesis conditions of nanotubes are well-controlled. The filled SWCNTs act as a catalyst source, carbon feedstock and container providing shielded environment for the tube growth at the same time. A fixed stoichiometry of metal to carbon atoms is achieved by the thermally-induced decomposition of organometallic molecule, and therefore the chemical composition of catalyst and carbon source is specified. The diameter of the outer SWCNTs defines the diameter of inner tubes, and thus it can be controlled by the choice of pristine SWCNT material.

### Investigation of growth dynamics of nanotubes

#### Nanotube growth in the CVD process

**Growth model of nanotubes.** Growth kinetics was actively studied for carbon nanotubes synthesized by the CVD method. The growth of nanotubes is characterized by the growth rate, which is their elongation rate during the synthesis process, and a growth time, which is the period of time during that the elongation of nanotubes occurs [23]. The quantity of nanotubes (a thickness of nanotube forests or length of individual tubes) increases with synthesis time until some saturation value [64,154–161]. The growth process is hindered because of several factors, which may not be mutually exclusive [162]. Among them are the diffusion limitation factor, when a gaseous carbon precursor is restricted from a catalyst by the increasing height of a nanotube forest [163–164], the catalyst lifetime factor, when the activity of the catalyst decreases as growth proceeds [154–155,159], and the factor of carbon overcoating on the surface of the metal catalyst from excessive gas-phase decomposition [160,165].

The catalyst lifetime-limited kinetics of the nanotube growth is a self-exhausting process that can be expressed by the following differential equation:

[2]
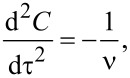


where *C* is the quantity of the grown nanotubes, τ is the synthesis time and ν is the lifetime of the catalyst [155]. After integration, Equation 2 is written in the form:

[3]
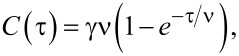


where *C*(τ) is the evolution of the quantity of nanotubes, γ is an initial growth rate of nanotubes. Many authors reported that this model fitted the observed growth curves of nanotube forests well [129–130,154–155,159,166–171].

**Figure 10:**
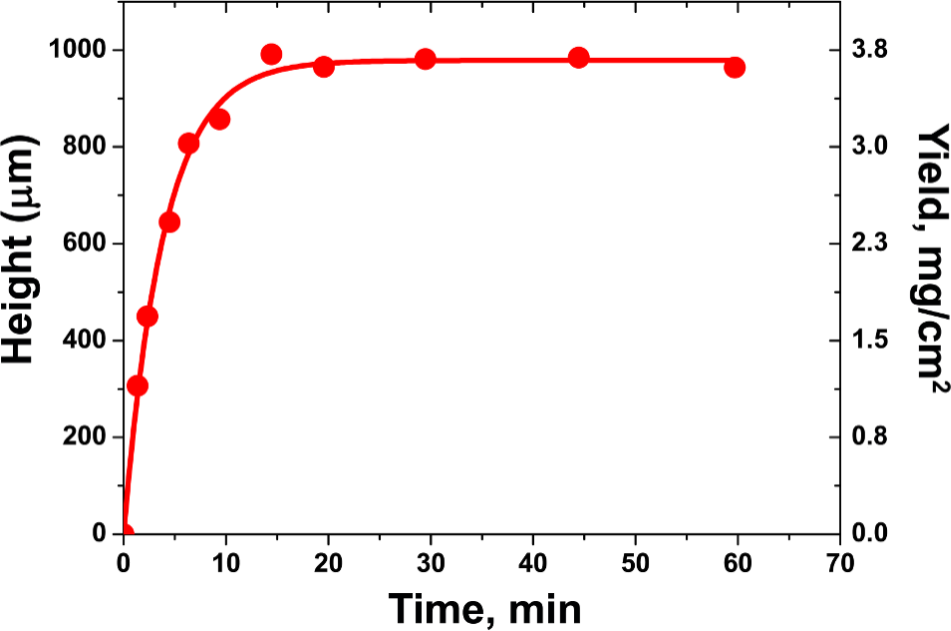
Time evolution of the height (yield) of SWCNT forest. Plot of the height of SWCNT forest as a function of the growth time. The experimental data (red circles) are presented together with the curve fitting using the Equation 2 (solid line). The data are replotted from [159].

Figure 10 demonstrates typical time evolution of the height (yield) of SWCNT forest at a fixed growth condition in the water-assisted CVD using C_2_H_4_ as a carbon source (the so-called “supergrowth” CVD) [159]. It shows that the growth rate is highest at the beginning of growth, gradually decreases over the subsequent 20 min and finally terminates with a height of 970 µm. The growth curve was fitted using the Equation 2. The fitting parameters are initial growth rate of nanotubes γ of 207 µm/min and catalyst lifetime ν of 4.74 min. The authors of [159] reported that similar behavior with varying terminal heights was observed on a number of time-evolution experiments that covered a broad range of growth conditions (growth temperature, C_2_H_4_ level and water level). Therefore, this behavior was regarded as a general feature of the supergrowth.

Some authors demonstrated that the growth dynamics did not follow a simple exponential model, because other factors than the catalyst decay or combination of several factors hindered the growth process [162,172–175].

**Growth rate of nanotubes.** The growth rate of nanotubes depends on the synthesis conditions: the pressure of gaseous carbon source, size of catalyst particles, chemical nature of catalyst and synthesis temperature. Table 2 summarizes the influence of the synthesis parameters on the growth rate of nanotubes.

**Table 2:** Dependence of the growth rate of nanotubes on synthesis parameters.

Synthesis parameter	Type of dependence	Reference

pressure of gaseous carbon source	growth rate increases with raising the pressure of carbon precursor	[154–155,159,161,164,175–180]
size of catalyst particles	growth rate increases with decreasing the size of catalyst particles	[97,181–185]
chemical nature of catalyst	no significant trend was revealed	[42,183,186–188]
synthesis temperature	growth rate increases nonlinearly with temperature	[42,82,154–155,160–162,164,166–168,174–175,177–180,183,187–204]

**Dependence of growth rate on pressure of carbon precursor.** Most studies reported on an increase of the growth rate of nanotubes with raising the pressure of gaseous carbon precursor: C_2_H_4_ [159,164,175–177], C_2_H_2_ [161,178–179], CH_4_ [180] and C_2_H_5_OH [154–155]. The same trend was reported in theoretical work [205]. This effect was explained by the increased amount of available carbon for the nanotube growth. On the basis of these data, it was concluded that the reaction of the nanotube growth could not be zero order. A linear dependence of the growth rate of nanotubes on the pressure of gaseous hydrocarbon testified that the reaction order was unity [164,175,178–180,205]. However, there are also reports where the reaction orders were estimated to be between 0 and 1 [161,176] and were observed to change with growth temperature [176]. Several authors reported that the growth rate was linearly proportional to the precursor pressure (first order reaction) until some critical value of pressure. Above this critical value, the growth rate became independent on the precursor pressure [154–155]. This was explained by a change of the kinetic regime of the nanotube growth from gas-phase diffusion limited to surface processes limited [155]. The authors of [177] observed linear dependences of the growth rate on precursor pressure with different slopes at low and high pressures. This was explained by the fact that at low precursor pressures the kinetic regime of the tube growth was surface diffusion limited and at high pressures – dissociation limited [177].

**Dependence of growth rate on size of catalyst particles.** Many authors reported that the growth rate of nanotubes increases with decreasing the size of catalyst particles. This trend was observed in the synthesis processes using different catalysts: Ni, Co, Fe [181], Co [97], Ni [182–183] and Fe [184–185]. For example, the authors of [183] showed that the growth rate of nanotubes in the CVD synthesis using nickelocene as catalyst precursor and C_2_H_2_ as carbon source increased by a factor of 3 while decreasing the size of Ni catalyst particles from 3.1 to 2.2 nm. This effect can be explained by the increased catalytic activity of smaller diameter particles due to their larger specific surface area, larger curvature of surface and, consequently, larger amount of active sites [23,206]. Indeed, larger catalytic activity for smaller particles is a commonly observed effect [207–209]. Several authors also related the increased catalytic activity of smaller particles for the nanotube growth to their modified electronic structure [23] as well as increased carbon solubility [183] and shortened diffusion length of carbon atoms to arrive at the growth site [182].

It was shown that the diameter of grown nanotubes is strongly correlated with the size of catalyst particles, i.e., smaller particles lead to the growth of smaller diameter nanotubes [64,97,182,186,202–204]. Consequently, many studies report that smaller diameter nanotubes have higher growth rates [97,182]. The nanotube growth rate was found to be inversely proportional to the tube diameter in [97,203]. Additionally, it was reported that coarsening the catalyst particles with increasing growth temperature of nanotubes led to the shift of their diameter distribution towards larger diameters [187,200–202,204,210]. For example, the authors of [200] showed that the average diameter of nanotubes synthesized by the thermal CVD method using C_2_H_2_ as carbon source increased from 20 to 150 nm while increasing growth temperature from 800 to 1100 °C.

**Dependence of growth rate on chemical nature of catalyst.** The chemical nature of the catalyst defines its chemical and physical properties and thus may influence the growth rate of nanotubes [23]. Several reports were dedicated to the comparison of kinetics of the growth of nanotubes in the CVD synthesis using different catalysts [42,183,186–188]. The authors of [186] performed a systematic study of the influence of Fe, Co and Ni catalysts on the growth of aligned nanotubes by the PECVD method. They found that the nature of catalyst has a strong effect on the diameter of nanotubes, their growth rate, wall thickness and morphology. Ni catalyst yielded the highest growth rate, largest diameter, thickest walls and cleanest wall surface of nanotubes, whereas Co catalyst resulted in the lowest growth rate, smallest diameter and thinnest walls of nanotubes covered with amorphous carbon. Similarly, it was shown in [42] that the growth rates of nanotubes on Ni and Co catalysts were very similar, whereas the rate on Fe catalyst was lower in the PECVD synthesis at temperatures of 250–500 °C. The authors of [183] demonstrated that the growth rate of nanotubes on Ni nanoparticles was about 2 times larger than the one on Fe particles with similar diameter in the thermal CVD synthesis using ferrocene and nickelocene as catalyst source and C_2_H_2_ as carbon source. Also, the growth rates of nanotubes varied for bimetallic (Ni/Fe) catalytic particles with different metal concentrations. These results were in agreement with the theoretical study [211] that predicted the increased growth rate of nanotubes on Ni catalyst as compared to the one on Fe catalyst due to faster integration of carbon into growing nanotubes. In contrast, the authors of [187–188] showed that the growth rate of nanotubes on Fe catalyst was about 2 times higher than the one on Co and Ni catalysts in the thermal CVD synthesis using C_2_H_2_ as carbon source at temperatures of 900–1000 °C [188] and in the pyrolysis of metal phthalocyanines at temperatures of 700–1000 °C [187]. This was explained by the fact that Fe is a more efficient metal in terms of carbon saturation than Co and Ni. Also, nanotubes grown on Fe catalyst had a better crystallinity of walls.

**Figure 11:**
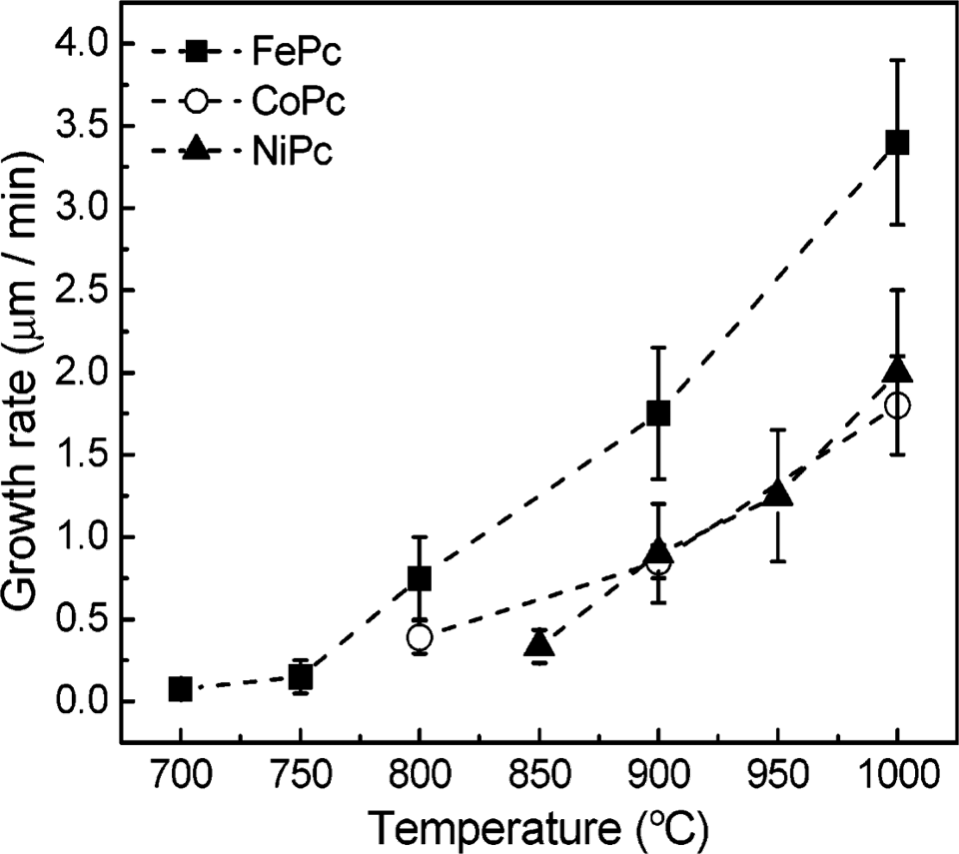
The plots of the growth rate as a function of synthesis temperature for the nanotubes grown via pyrolysis of iron, nickel and cobalt phthalocyanines. Reprinted with permission from [187], copyright 2003 American Chemical Society.

**Dependence of growth rate on synthesis temperature.** All studies dedicated to the investigation of the dependence of the growth rate of nanotubes on temperature reported that the rate increased nonlinearly with temperature [42,82,154–155,160–162,164,166–168,174–175,177–180,183,187–204]. For example, the authors of [200] found that the growth rate of nanotubes increased exponentially from 1.6 to 28 µm/min (by a factor of 18) while increasing the growth temperature from 800 to 1100 °C in the thermal CVD process using Fe as catalyst and C_2_H_2_ as carbon source. Similarly, the authors of [187] found that the growth rate of nanotubes increased exponentially from 0.075 to 3.5 µm/min (by a factor of 47) while increasing the growth temperature from 700 to 1000 °C in the pyrolysis process using iron phthalocyanine as catalyst and carbon source. Figure 11 shows the obtained plots of the growth rate as a function of synthesis temperature for the nanotubes grown via pyrolysis of iron, nickel and cobalt phthalocyanines [187]. They show a nonlinear increase of the growth rate with temperature. The growth rate of nanotubes using iron phthalocyanine is about 2 times higher than in the case of nickel and cobalt phthalocyanines. These results are in agreement with studies on the growth of carbon filaments, where the growth rates also increased exponentially with temperature [19–20,181,212–213].

This behavior is caused by the fact that the catalytic nanotube growth is a thermally-activated process. The dependence of the growth rate on temperature obeys the Arrhenius equation [214]:

[4]
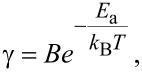


where γ is the growth rate of nanotubes, *E*_a_ is the activation energy of the nanotube growth, *k*_B_ is the Boltzmann constant, *T* is the absolute temperature and *B* is a proportionality coefficient.

**Activation energy of nanotube growth.** Many studies reported the calculation of activation energies of the nanotube growth, taking into consideration the Arrhenius equation (Equation 4). Indeed, if we take the natural logarithm from both parts of Equation 4, we get the following expression:

[5]
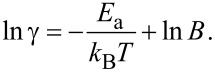


According to Equation 5, the natural logarithm of the growth rate shows a linear dependence on the inverse growth temperature. The slope of this linear dependence is −*E*_a_/*k*_B_. Thus, the linear fitting of the dependence ln γ(1/*T*) yields directly the value of the activation energy of the nanotube growth.

**Figure 12:**
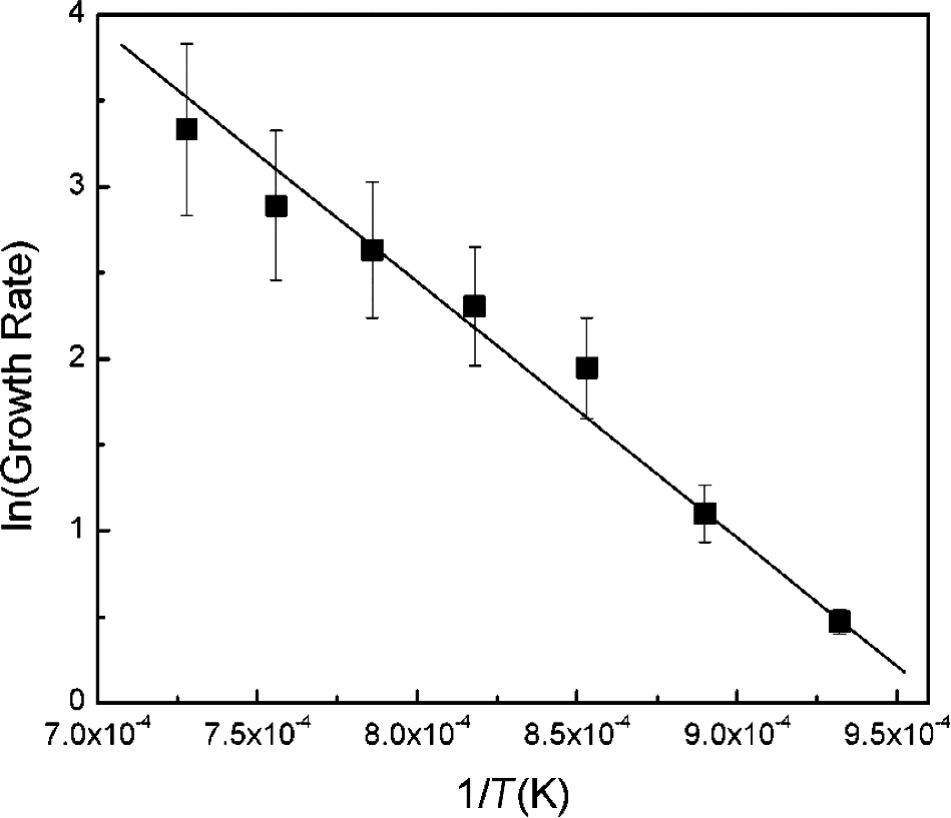
The Arrhenius plot for the growth rates of MWCNTs synthesized by thermal CVD using C_2_H_2_ as carbon source and Fe catalyst at 800–1100 °C. The experimental data (black squares) are shown together with linear fitting, providing the activation energy of 1.3 eV. Reprinted with permission from [200], copyright 2002 American Chemical Society.

Figure 12 shows an example of the Arrhenius plot for the growth rates of MWCNTs synthesized by thermal CVD using C_2_H_2_ as carbon source and Fe catalyst at 800–1100 °C in [200]. The experimental data fit well to a linear function, providing the activation energy of 1.3 eV.

The activation energies calculated in the literature vary in the range from 0.1 to 2.8 eV. Table 3 summarizes the activation energies of the nanotube growth by different synthesis methods using various carbon precursors and catalysts in a chronological order.

**Table 3:** Summary of reports dedicated to the investigation of growth dynamics of nanotubes. Given are the type of synthesized nanotubes, synthesis conditions, calculated activation energy of the nanotube growth and assigned growth rate-limiting process (together with the reported activation energy for this process) in a chronological order.

Type of synthesized nanotubes	Method of synthesis	Source of carbon	Catalyst/support	Synthesis temperature	Calculated activation energy of nanotube growth	Assigned growth-rate-limiting process, reported activation energy for this process	Ref.

carbon filaments	catalytic thermal decomposition	C_2_H_2_	Ni (30–50 nm)/ support	≈600 °C	1.51 eV	bulk diffusion of carbon through the solid catalyst particle (1.43–1.51 eV [215])	[19]
carbon filaments	catalytic thermal decomposition	C_2_H_2_	α-Fe/support (graphite, silicon)	≈600 °C	0.70 eV	bulk diffusion (0.46–0.72 eV [216–217])	[20]
			Co/support (graphite, silicon)	≈600 °C	1.44 eV	bulk diffusion (1.51 eV [218])	
carbon filaments	catalytic thermal decomposition	C_2_H_2_	V (50 nm)/ graphite	600–825 °C	1.20 eV	bulk diffusion (1.21 eV [219])	[212]
			Mo (10–25 nm)/ graphite	445–680 °C	1.68 eV	bulk diffusion (1.78 eV [220])	
carbon filaments	catalytic thermal decomposition	C_2_H_2_	α-Fe/silica	530–900 °C	0.79 eV	bulk diffusion	[213]
			γ-Fe (20 nm)/ graphite	380–685 °C	1.47 eV	bulk diffusion (1.45–1.62 eV [221–222])	
carbon filaments	catalytic thermal decomposition	1,3-butadiene (C_4_H_6_) + H_2_ + Ar	Ni (10–30 nm)/ Al_2_O_3_	400–800 °C	1.35–1.55 eV	bulk diffusion of carbon through the solid catalyst particle	[85]
VA tubular MWCNT (20–30 walls)	PECVD	C_2_H_2_ + NH_3_	Ni (or Co) thin film (0.5–20 nm)/Si with SiO_2_ layer	500–900 °C	0.56 eV	surface diffusion of carbon across the catalyst particle	[203]
SWCNT	laser ablation	Graphite target	0.6 atom % Ni + 0.6 atom % Co	850–1250 °C	0.38 eV	carbon diffusion through the molten catalytic particle	[189]
randomly oriented MWCNT (*d* = 5–100 nm)	thermal CVD	C_2_H_2_ + NH_3_	Ni thin film (3 nm)/Si with SiO_2_ layer	550–850 °C	1.21 eV	bulk diffusion of carbon through the solid catalyst particle	[204]
VA bamboo-like CNT (*d* = 30–100 nm)	PECVD				0.76 eV	surface diffusion of carbon across the catalyst particle	
VA bamboo-like MWCNT (*d* = 20–150 nm)	thermal CVD	C_2_H_2_	Fe/Si	800–1100 °C	1.30 eV	bulk diffusion of carbon through the solid catalyst particle	[200]
VA bamboo-like CNT	PECVD	C_2_H_2_ + NH_3_	Ni thin film (6 nm)/Si with SiO_2_ layer	120–550 °C	0.23 eV	surface diffusion of carbon across the solid catalyst particle (0.3 eV [223])	[190]
VA MWCNT (*d* =10–120 nm)	pyrolysis	Fe, Co and Ni phthalocyanines (+ Ar + H_2_, SiO_2_ substrate)		700–1000 °C	1.30 eV	bulk diffusion of carbon through the solid catalyst particle (*E*_a_(γ-Fe) = 1.52 eV, *E*_a_(Co) = 1.61 eV, *E*_a_(Ni) = 1.43 eV [181,224])	[187]
VA MWCNT (*d* =10–30 nm)	pyrolysis	C_2_H_2_ + ferrocene (+Ar)		700–1000 °C	1.52 eV	bulk diffusion of carbon through the solid catalyst particle	[191]
MWCNT	catalytic thermal decomposition	C_2_H_2_ + N_2_ + H_2_	Fe/SiO_2_	600–800 °C	1.79 eV	bulk diffusion of carbon through the solid catalyst particle	[82]
tubular MWCNT (*d* ≈ 10 nm)	microwave CVD	CH_4_ + H_2_	Fe (or Co, or Ni) thin film (2 nm)/Si with SiO_2_ layer	900–1100 °C	0.32 eV (Fe), 0.32 eV (Co), 0.55 eV (Ni)	bulk diffusion of carbon through the molten catalyst particle	[192]
bamboo-like CNT (*d* ≈ 10 nm)				800–950 °C	1.4 eV (Fe), 1.5 eV (Co), 1.6 eV (Ni)	bulk diffusion of carbon through the solid catalyst particle	
VA MWCNT (*d* = 10–20 nm)	thermal CVD	C_2_H_2_ + ferrocene (+Ar, Si with SiO_2_ substrate)		600–800 °C	1.30 eV	bulk diffusion of carbon through the solid catalyst particle	[193]
VA MWCNT	thermal CVD	C_2_H_2_ + Ar	Fe thin film (3–5 nm)/Si with SiO_2_ layer	600–727 °C	1.65 eV	surface reaction at the gas–catalyst interface (*E*_a_(heterogeneous decomposition of C_2_H_2_) = 1.86 eV at 352–472 °C [225] and 1.13 eV at 1060–1255 °C [226])	[178]
carbon nanofiber (*d* ≈ 50 nm)	PECVD	C_2_H_2_ + NH_3_	Ni (or Co, or Fe) thin film (5–15 nm)/Si with SiO_2_ layer	120–500 °C	0.23 eV (Ni), 0.30 eV (Co), 0.35 eV (Fe)	surface diffusion of carbon on the catalyst particle	[42]
VA MWCNT, DWCNT or SWCNT	thermal CVD	C_2_H_2_ + H_2_ + Ar	Fe (1 nm) + Mo (0.2 nm) thin films/Al (10 nm)/Si	535–900 °C	2.2 eV	contribution of multiple chemical processes input into activation energy	[160]
SWCNT (*d* = 0.6–3.5 nm)	catalytic thermal decomposition (inside UHV TEM)	C_2_H_2_	Ni (<6 nm)/MgO	650 °C	2.7 eV (nucleation barrier for carbon adatoms to form the hemispherical graphene cap)	formation of a hemispherical graphene cap on the catalyst particle	[64]
MWCNT (*d* ≈ 15 nm)	thermal CVD	C_2_H_2_ or C_2_H_4_ + H_2_	Ni nanoparticles (≈15 nm) generated in the pulsed laser ablation particle source	400–600 °C	0.80 eV (C_2_H_2_) 0.83 eV (C_2_H_4_)	both surface diffusion and bulk diffusion of carbon through the catalyst particle	[194]
small diameter (3–10 nm) MWCNT	thermal CVD with a fixed bed flow reactor	CH_4_ + N_2_	Mo_x_Co_y_– Mg_1−x−y_O	650–800 °C	1.55–1.69 eV	Decomposition of gaseous carbon source (0.69 eV [227])	[180]
large diameter (10–30 nm) MWCNT			Co_x_Mg_1−x_O	550–650 °C	1.00 eV		
bamboo-like CNT	catalytic thermal decomposition (inside UHV TEM)	C_2_H_2_	Ni (7–30 nm)/ MgO	650 °C	2.91 eV (nucleation barrier for C adatoms to form the circular cap)	formation of a hemispherical cap on the catalyst particle	[60]
VA MWCNT	thermal CVD	C_2_H_4_ + H_2_ + Ar + H_2_O	Fe layer (1.5 nm)/Al_2_O_3_ (10 nm) layer on Si	670–710 °C	2.09 eV	surface reaction	[164]
VA MWCNT (*d* = 10–20 nm)	thermal CVD	C_2_H_4_	Fe thin film (2.5 nm)/Si with SiO_2_ or Al_2_O_3_ barrier layers	600–700 °C	2.00 eV (with Al_2_O_3_) 2.16 eV (with SiO_2_)	contribution of multiple chemical processes input into activation energies	[195]
MWCNT (*d* ≈ 10 nm)	thermal CVD	C_2_H_2_ + H_2_	Ferrocene or nickelocene (produced Fe or Ni particles of ≈3 nm size inside microplasma reactor)	475–605 °C	1.21 eV (Fe) 0.76 eV (Ni)	surface diffusion of carbon on the catalyst particle	[196]
MWCNT	thermal CVD	C_2_H_4_ + H_2_ + He	Fe–Co/Al_2_O_3_	600–700 °C	1.35 eV	elimination of the first atom of hydrogen from the adsorbed ethylene	[176]
VA SWCNT	thermal CVD	C_2_H_5_OH	(Co,Mo)/quartz	750–825 °C	1.5 eV	bulk diffusion of carbon through the solid catalyst particle	[154]
VA MWCNT	thermal CVD	xylene + ferrocene (+H_2_ + Ar, conductive substrate)		500–820 °C	1.41 eV	bulk diffusion of carbon through the catalyst particle	[174]
MWCNT (*d* ≈ 10 nm)	thermal CVD	C_2_H_2_ + H_2_	ferrocene and nickelocene (produced Ni, Fe or NiFe particles of ≈3–4 nm size inside microplasma reactor)	400–600 °C	0.76 eV (Ni) 0.57 eV (Ni_0.88_Fe_0.12_) 0.38 eV (Ni_0.67_Fe_0.33_) 0.42 eV (Ni_0.27_Fe_0.73_) 1.23 eV (Fe)	both surface diffusion and bulk diffusion of carbon through the catalyst particle	[183]
SWCNT	thermal CVD	C_2_H_5_OH	Ni (or Co) thin film/Si with SiO_2_	500–900 °C	2.8 eV (Ni) 2.4 eV (Co) (at temperatures of 500–580 °C)	catalytic decomposition of the carbon precursor (*E*_a_(decomposition of ethanol into ethylene) = 2.7 eV [228])	[155]
VA SWCNT	thermal CVD	C_2_H_5_OH	Co thin film (0.8 nm)/Al_2_O_3_ (250 nm)/Si with SiO_2_	650–1000 °C	1.1 eV (at temperatures of 650–870 °C)	bulk diffusion of carbon through the catalyst particle	[166]
VA MWCNT or SWCNT	thermal CVD (atmospheric or low pressure)	C_2_H_2_ + H_2_ + Ar	Fe thin film (0.5–1 nm)/Al_2_O_3_ (10 nm)/Si with SiO_2_	560–800 °C	0.95 eV (14 mbar C_2_H_2_) 0.93 eV (0.37 mbar C_2_H_2_) 0.98 eV (10^−3^ mbar C_2_H_2_)	bulk diffusion of carbon through the catalyst particle	[161]
VA MWCNT (*d* ≈ 10 nm)	decoupled thermal CVD (with preheating of gaseous carbon source)	C_2_H_4_ + H_2_ + He	Fe thin film (1 nm)/Al_2_O_3_ (10 nm)/Si with SiO_2_	900–1120 °C (preheating, *T*_p_) 675–875 °C (CVD)	1.02 eV (*T*_p_ = 980 °C) 1.28 eV (*T*_p_ = 1020 °C) 1.44 eV (*T*_p_ = 1070 °C) 1.88 eV (*T*_p_ = 1120 °C)	cumulative process of gaseous carbon source decomposition and rearrangement	[162]
VA MWCNT	thermal CVD (with preheating of gaseous carbon source)	C_2_H_4_ + H_2_ + Ar	Fe thin film on conductive metallic substrate	650–750 °C (preheating) 475 °C (substrate)	0.9 eV	thermal decomposition of gaseous carbon source	[197]
				no preheating 475–600 °C (substrate)	0.1 eV		
MWCNT (*d* ≈ 15–30 nm)	thermal CVD with a fluidized bed reactor	C_2_H_2_ + H_2_ + N_2_	Fe (or Ni)/mesoporous Al_2_O_3_ (specific surface area of 157 m^2^/g)	700–850 °C	0.68 eV (Ni) 0.27 eV (Fe)	not assigned	[198]
MWCNT (*d* ≈ 20–30 nm)	thermal CVD with a fluidized bed reactor	C_2_H_2_ + H_2_ + N_2_	Ni (or Co)/CaCO_3_ (particle size of 100 µm)	700–850 °C	1.08 eV (Ni) 0.64 eV (Co)	not assigned	[179]
VA SWCNT + MWCNT	thermal CVD	C_2_H_4_ + H_2_ + He	Fe thin film (2 nm)/Al_2_O_3_ (30 nm)/Si	750–850 °C	2.6 eV	gas phase reaction that generates active precursors for the nanotube synthesis	[175]
VA SWCNT	water-assisted thermal CVD	C_2_H_4_ + H_2_ + He (+H_2_O)	Fe thin film (1 nm)/Al_2_O_3_ (10 nm)/Si	750–850 °C	2.83 eV	not assigned	[167]
VA MWCNT	laser-assisted CVD	C_2_H_4_ + H_2_ + Ar	Fe thin film (1.5 nm)/Al_2_O_3_ (20 nm)/Si	600–1000 °C	0.76 eV (Ar/C_2_H_4_/H_2_ = 200/25/50 sccm)	surface diffusion of carbon on the catalyst particle	[177]
					0.57 eV (Ar/C_2_H_4_/H_2_ = 200/250/50 sccm)	dissociation of gaseous carbon source into carbon	
					0.25 eV (Ar/C_2_H_4_/H_2_ = 500/10/50 sccm)	adsorption of gaseous carbon source on the catalyst particle	
					0.36 eV (Ar/C_2_H_4_/H_2_ = 10/10/50 sccm)	mass diffusion of gaseous carbon source	
MWCNT	thermal CVD with a fixed bed reactor	C_2_H_4_ + H_2_ + Ar	Co–Mn–Al–Mg mixed oxide catalyst (pore size of 4–8 nm, specific surface area of ≈130 m^2^/g)	600–700 °C	1.11 eV	possible influence of mass transfer phenomena inside catalyst particles on the effective reaction rate	[199]
VA SWCNT	water-assisted thermal CVD	C_2_H_4_ or C_2_H_2_ or C_4_H_10_ or C_3_H_8_ + He (+H_2_O)	Fe thin film (1.8 nm)/Al_2_O_3_ (40 nm)	725–825 °C	1.0–2.8 eV (C_2_H_4_, carbon concentration in reacting gas mixture varies from 10 to 3%)	Increasing carbon concentration changes the rate-limiting process from gas dissociation/ adsorption on the catalyst to bulk diffusion of carbon through the catalyst particle	[168]
					1.9–2.4 eV (C_4_H_10_, carbon concentration in reacting gas mixture varies from 4 to 8%)	At all carbon concentrations, the rate limiting process is gas dissociation/ adsorption on the catalyst	

Baker with co-authors performed the first calculations of the activation energies of the growth of carbon filaments by the catalytic thermal decomposition of C_2_H_2_ on different catalysts in 1970–1980s: Ni [19], Co [20], Fe [20,213], V [212] and Mo [212]. The obtained values (0.7–1.68 eV) were similar to the activation energies for the solid-state carbon diffusion through the corresponding bulk metals. On the basis of these data, it was concluded that the bulk diffusion through the solid-state catalyst particle was the growth rate-limiting process. The same correlation was reported in [85], where the activation energy of the growth of carbon filaments by the catalytic thermal decomposition of 1,3-butadiene on Ni was estimated to range from 1.35 to 1.55 eV. Similar values and explanations were reported by other authors for the growth of carbon nanotubes. The activation energies of the MWCNT growth were calculated to be 1.3 eV [200], 1.79 eV [82] and 1.21 eV [204] for the thermal CVD synthesis using C_2_H_2_ as carbon source and Fe or Ni catalysts, 1.52 eV [191] and 1.30 eV [193] for the thermal CVD synthesis using C_2_H_2_ as carbon source and ferrocene as precursor of catalyst, 1.30 eV for the pyrolysis of Fe, Co and Ni phthalocyanines [187] and 1.41 eV for the thermal CVD synthesis using xylene as carbon source and ferrocene as precursor of catalyst [174]. The authors of [154,166] found that the activation energy of SWCNT growth by the thermal CVD synthesis using C_2_H_5_OH as carbon source equaled 1.5 eV for Co-Mo catalyst [154] and 1.1 eV for Co catalyst [166]. In [161], the activation energy of the growth of a mixture of MWCNTs and SWCNTs by the thermal CVD synthesis using C_2_H_2_ and Fe catalyst was estimated to be 0.93–0.98 eV for a broad range of C_2_H_2_ pressures (from 10^−3^ to 14 mbar).

The bulk diffusion was also proposed as the growth rate-limiting process in the laser-ablation growth of SWCNTs using a graphitic target with Ni–Co catalyst [189]. However, low activation energy (0.38 eV) testified that carbon was diffused through the molten catalyst particle. Indeed, the laser ablation process was conducted at higher temperatures (up to 1250 °C) than usually used in the thermal CVD synthesis. The authors of [192] found that in the microwave CVD process using CH_4_ as carbon source and metallic (Fe, Co, Ni) catalysts, the bamboo-like MWCNTs were synthesized at temperatures of 800–950 °C and tubular MWCNTs at 900–1100 °C. The activation energies of the growth of bamboo-like MWCNTs were estimated to be 1.4 (Fe), 1.5 (Co) and 1.6 eV (Ni), whereas the values for the tubular MWCNTs were 0.32 (Fe and Co) and 0.55 eV (Ni). The observed differences were explained by the fact that bulk diffusion was the growth rate-limiting process in both cases, but through solid or molten catalyst particle at different growth temperatures.

Much lower activation energies of the nanotube growth were observed in the PECVD synthesis. In [190], the activation energy of the MWCNT growth in the process using C_2_H_2_ as carbon source and Ni catalyst at low temperatures (120–550 °C) was estimated to be 0.23 eV. This value was close to the activation energy of surface diffusion of carbon on polycrystalline Ni. On the basis of these data, it was concluded that the diffusion of carbon on the catalyst surface was the growth rate-limiting step at low temperatures. The use of plasma in the synthesis process increased the dissociation of C_2_H_2_. At low temperatures, the solubility of carbon in Ni was low and thus the bulk diffusion of carbon was limited. However, carbon atoms adsorbed on the surface of the catalyst particle could diffuse across the surface much faster [190]. The similar values of activation energies were calculated by the authors of [42] in the PECVD synthesis using C_2_H_2_ and metallic (Fe, Co, Ni) catalysts: 0.23 (Ni), 0.30 (Co) and 0.35 eV (Fe). The authors of [203–204] synthesized MWCNTs by the PECVD method using C_2_H_2_ with Ni and Co catalysts at higher temperatures (500–900 °C) and obtained slightly higher values: 0.56 eV [203] and 0.76 eV [204]. However, they were also attributed to the surface diffusion of carbon on catalyst particles.

The intermediate values of activation energies between bulk and surface carbon diffusion energies obtained in [183,194,196] were attributed to the contribution of both these processes in the nanotube growth. The authors of [194] found that the activation energy of MWCNT growth by the thermal CVD process on Ni catalyst did not depend on the carbon source: the value equaled 0.80 and 0.83 eV for C_2_H_2_ and C_2_H_4_, respectively. In [183,196], it was demonstrated that the activation energy of MWCNT growth by the thermal CVD method using C_2_H_2_ as carbon source depended on the used catalyst. It equaled 1.21 eV for Fe, 0.76 eV for Ni and 0.38–0.57 eV for bimetallic Ni–Fe catalysts.

Several authors found that the reaction at the gaseous carbon source–catalyst interface was the rate-limiting process in the nanotube growth [155,162,164,175–176,178,180,197]. In these reports, the calculated activation energies were usually larger than the values for bulk carbon diffusion in metals. For example, the authors of [164] estimated the activation energy of the MWCNT growth by the thermal CVD method using C_2_H_4_ as carbon source and Fe catalyst at 670–710 °C to be 2.09 eV. The authors of [175] calculated the activation energy of the growth of the mixture of SWCNTs and MWCNTs of 2.6 eV in the similar process conducted at higher temperatures (750–850 °C). In [155], the estimated activation energy of SWCNT growth by the thermal CVD method using C_2_H_5_OH as carbon source and Ni or Co catalysts depended on the nature of catalyst. It equaled 2.8 eV for Ni and 2.4 eV for Co. These values were similar to the activation energy of the decomposition of ethanol into ethylene.

The authors of [162,197] showed that the activation energy of the nanotube growth depended strongly on the pre-treatment of gaseous carbon source. In [162], the carbon precursor (C_2_H_4_) was pre-heated at temperatures of 980–1120 °C before introducing into the CVD reactor with Fe catalyst on a substrate heated up to 675–875 °C. The calculated activation energy of the nanotube growth depended on the pre-heating temperature of carbon precursor: the value increased from 1.02 to 1.88 eV with increasing temperature from 980 to 1120 °C. On the basis of these data, it was suggested that the cumulative process of gaseous carbon source decomposition and rearrangement was the rate-limiting step. The authors of [197] compared the activation energies of the MWCNT growth in the thermal CVD process using C_2_H_4_ and Fe catalyst with and without preheating of carbon source. Without preheating of the carbon precursor, the nanotubes grew at temperatures of substrate of 475–600 °C with the activation energy of 0.1 eV. When the carbon precursor was pre-heated at temperatures of 650–750 °C, the nanotubes grew at temperature of substrate of 475 °C with the activation energy of 0.9 eV.

In recent reports [168,177], it was demonstrated that varying the concentration of gaseous carbon precursor in the reacting gas mixture may lead to changes of the activation energy of the nanotube growth due to switching between different growth rate-limiting processes. The authors of [177] performed the growth of MWCNTs by the laser-assisted CVD method using C_2_H_4_ as carbon source (Ar and H_2_ were used as gas carriers) and Fe catalyst at temperatures of 600–1000 °C. Varying the concentration of C_2_H_4_ allowed changing the activation energy between the values of 0.25 eV, which was assigned to the adsorption of gaseous carbon source on the catalyst particle, 0.36 eV, which corresponded to the mass diffusion of the carbon source, 0.57 eV, which was assigned to the dissociation of the carbon precursor to carbon, and 0.76 eV, which corresponded to the surface diffusion of carbon on the catalyst particle. In [168], the SWCNTs were grown by the water-assisted thermal CVD method using C_2_H_4_ or C_4_H_10_ as carbon source (in the mixture with He and H_2_O) and Fe catalyst at 725–825 °C. When C_2_H_4_ was used, the activation energy of the nanotube growth decreased from 2.8 to 1.0 eV with increasing the carbon concentration in the reacting gas mixture from 3 to 10%. This was explained by the fact that the growth rate-limiting process switched from the carbon precursor dissociation/adsorption on the catalyst to the bulk diffusion of carbon through the catalyst particle. When C_4_H_10_ was used, the activation energy increased from 1.9 to 2.4 eV with increasing the carbon concentration in the initial gas mixture from 4 to 8%. This was explained by the fact that the growth rate-limiting step was the gaseous carbon source dissociation/adsorption on the catalyst at all carbon concentrations.

**Lifetime of catalyst.** The authors of reports on the nanotube growth where growth kinetics employed a first order exponential model of the catalyst decay demonstrated that the lifetime of catalyst depended on the pressure of gaseous carbon precursor and growth temperature. In [159], it was shown that an increase of the C_2_H_4_ pressure in the water-assisted thermal CVD synthesis of SWCNTs on Fe catalyst led to a gradual decrease of the lifetime of the catalyst. The authors of [168] demonstrated that the lifetime evolution with changing precursor pressure depended on the used carbon feedstock. An increase in the C_2_H_4_ and C_4_H_10_ pressures in the thermal CVD synthesis with Fe catalyst caused a decrease and increase of the lifetime, respectively, and it pointed out different rate-limiting processes of the nanotube growth. Using another carbon precursor (C_2_H_5_OH) and catalysts (Ni and Co), the authors of [155] found that the lifetime decreased with increasing the precursor pressure. Additionally, they showed that the lifetime decreased with increasing growth temperature until a critical temperature, above which it increased with temperature. The authors of [166,168,195] reported a decrease of the lifetime with increasing the growth temperature while using different carbon precursors and catalysts.

The correlation between the growth rate of nanotubes and lifetime of catalyst was discussed [155,159,166,168,195]. In [159,195], the values were found to be inversely correlated: the lifetime increased while the growth rate decreased and vice versa. In [155,166], it was reported that the evolution of the growth rate and lifetime with temperature was opposite in a limited range of temperatures. The authors of recent publication [168] performed a systematic study of the relationship between the growth rate and lifetime for over 300 SWCNT forests synthesized by the thermal CVD method using different carbon precursors (C_2_H_2_, C_2_H_4_, C_4_H_10_ and C_3_H_8_), carbon concentrations and growth temperatures. In all cases, they found an inverse relationship between the growth rate of nanotubes and lifetime of catalyst. On the basis of these data, they suggested that this dependence is a fundamental phenomenon that stems from the growth mechanism of nanotubes.

The initial growth rate of SWCNTs in the CVD synthesis is in the order of tens µm/min and the growth time is in the order of tens of minutes. Depending on the growth rate and growth time of nanotubes, the reported synthesis procedures of SWCNT forests can be classified into two groups [167]. The first group includes processes with low growth rates, long lifetimes of catalysts and long growth times of nanotubes. The long synthesis was conducted by the microwave plasma CVD method with an initial rate of ≈2.6 µm/min for ≈32 h, and it led to 5 mm SWCNT forests [229]. Also, long growth of SWCNTs by the water-assisted CVD method with a rate of 1.5 µm/min for 6 h was reported [230]. The second group includes processes with high growth rates, short lifetimes of catalysts and short growth times of nanotubes. For example, the water-assisted CVD synthesis of SWCNTs (called “supergrowth”) was conducted with a rate of ≈200 µm/min for ≈20 min [159]. Recently, the authors of [167] managed to increase the growth rate of SWCNTs up to 620 µm/min in the water-assisted CVD process with a growth time of 10 min.

#### Inner tube growth inside filled SWCNTs

**Growth properties of inner tubes inside fullerene-filled SWCNTs.** The first attempt to investigate the growth properties of inner tubes was made in 2004 [134]. The authors of [134] traced the time evolution of intensities of inner tube peaks in the RBM band of Raman spectra of fullerene C_60_-filled SWCNTs annealed at temperatures between 800 and 1200 °C for up to 250 h. They observed that inner tubes grew faster at higher temperatures. A clear difference between the growth curves of inner tubes with diameters smaller than ≈0.7 nm and those with larger diameters was revealed. For inner tubes with diameters ≤0.7 nm, the peak intensities increased in the beginning of annealing and then the rates became flattened. In contrast, for inner tubes with diameters ≥0.7 nm, the peak intensities kept growing. On the basis of these data, the growth model of inner tubes was suggested. In the beginning of annealing, adjacent C_60_ molecules polymerize and form inner tubes with a diameter close to that of C_60_ (≈0.7 nm). After that, the inner tubes increase their diameter to adjust the spacing between the inner and outer carbon shells to fit the van der Waals distance. As a result, the amount of the ≈0.7 nm diameter inner tubes is decreased and the amount of larger diameter tubes is increased with the annealing time.

**Figure 13:**
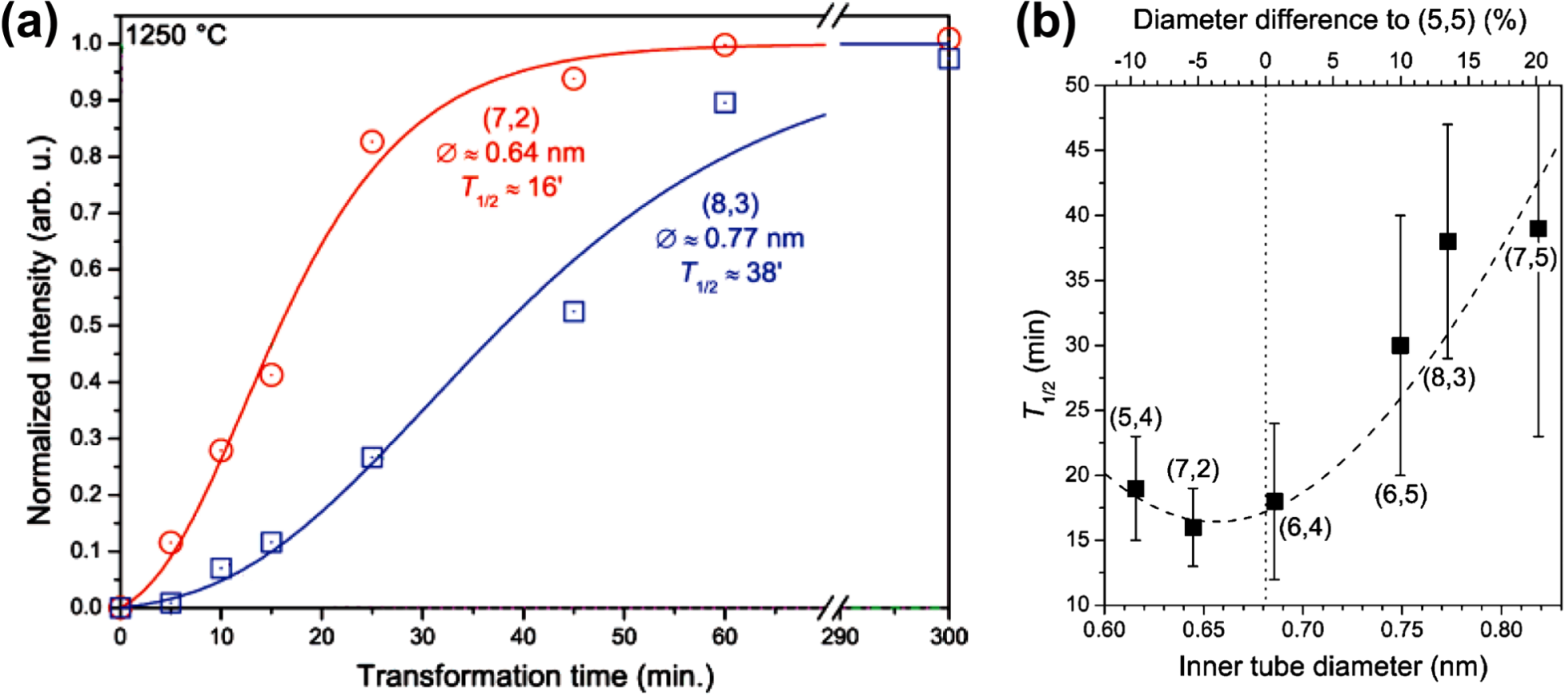
(a) The plot of the RBM peak intensities as a function of transformation time for the (7,2) and (8,3) inner tubes. The experimental data (circles and squares) are shown together with the fitting curves. The intensities for each tube were normalized to the respective maxima of the fitting curves. The tube diameter and estimated growth half-times of the tubes are indicated. (b) The growth half-time as a function of the tube diameter for the (5,4), (7,2), (6,4), (6,5), (8,3) and (7,5) inner tubes. The dotted line indicates the diameter of the (5,5) tube, which is the same as for C_60_ fullerene molecule. The dashed curve is a guide for the eye. Reprinted with permission from [140], copyright 2007 American Chemical Society.

The authors of [140] performed a detailed investigation of growth dynamics of inner tubes inside fullerene C_60_-filled SWCNTs. They monitored the increase of the peak intensities of inner tubes in the RBM-band of Raman spectra of the filled SWCNTs annealed at 1250 °C for up to 300 min. Figure 13a demonstrates the plot of the RBM peak intensities as a function of transformation time for the (7,2) tube with a diameter of 0.64 nm and (8,3) tube with a diameter of 0.77 nm. It is clearly visible that the growth of the smaller diameter (7,2) tube starts much earlier than the growth of the (8,3) tube, and it also saturates much earlier. The growth half-times of the (7,2) and (8,3) tubes were estimated to be 16 and 38 min, respectively. Using different excitation laser wavelengths, the authors of [140] also analyzed the growth dynamics of other inner tubes with chiralities of (5,4), (6,4), (6,5) and (7,5) and diameters ranging between 0.61 and 0.82 nm. The growth half-time of these nanotubes was in the range from 16 to 38 min. Figure 13b presents the plot of the growth half-time as a function of the inner tube diameter. It was found that the nanotubes with a diameter close to that of C_60_ (the (7,2) and (6,4) tubes) grew most rapidly, and the growth time increased with increasing the inner tube diameter. These results were in agreement with the above-discussed data reported in [134]. However, the authors did not observe that the amount of smaller diameter inner tubes decreased while the amount of larger diameter tubes increased. Therefore, they did not support the growth model of inner tubes proposed in [134] that small inner tubes were transformed into large tubes with increasing annealing time.

In [137], fullerene C_60_-filled SWCNTs were converted into DWCNTs by laser and furnace annealings, and the evolution of the transformation process was studied by Raman spectroscopy as a function of the laser power and annealing temperature. In the case of laser annealing, the power of 1064 nm laser was successively increased from 100 to 500 mW at a fixed annealing time of 1 min. The intensity of the RBM peaks of inner tubes increased with increasing laser power and reached the maximum at a power of 260 mW. At further increase of laser power, the amount of inner tubes decreased until a complete removal at 500 mW. In the case of furnace annealing, temperature was successively increased from 1000 to 1550 °C at a fixed annealing time of 1 h. The RBM peaks of inner tubes appeared at temperature of 1250 °C, and their intensity increased gradually with increasing temperature. The smaller diameter inner tubes were formed at lower temperatures than larger diameter ones. These finding was in line with the data reported in [134]. Nevertheless, the authors did not confirm the growth model suggested in [134]. They proposed that the diameter of inner tubes was fully determined by the diameter of the outer nanotubes and it stayed constant during annealing.

In recent theoretical study [231], the mechanisms of fullerene coalescence and transformations of sp^2^ carbon network to grow inner tubes were studied. A key step of such transformation was shown to be a rotation of a C–C bond in a sp^2^ carbon network (called the Stone–Wales transformation). The growth of inner tubes occurred though the cooperative motion of Stone–Wales defects, and it led to a preferential formation of tubes with high chiral angles and the abundance of metallic armchair tubes in the inner walls of the formed DWCNTs.

**Growth properties of inner tubes inside SWCNTs filled with organometallic molecules.** The authors of [232] investigated the temperature-dependent inner tube growth inside SWCNTs filled with ferrocene molecules. The evolution of the Raman spectra of the filled nanotubes with increasing annealing temperature from 500 to 1300 °C at a fixed growth time of 2 h was traced. It was found that inner tubes start to be formed at temperature of 500 °C and they grew rapidly with increasing temperature. Small diameter inner tubes (≈0.5 nm) were stable only until 1000 °C, whereas larger diameter tubes (≈1 nm) were not destructed until 1300 °C. This was explained by a higher reactivity of smaller diameter inner tubes towards oxidation.

In [233], the inner tubes were formed by laser annealing of ferrocene-filled SWCNTs using a 532 nm laser. The dependence of the inner tube growth on the laser power at a fixed annealing time of 1 min was investigated by Raman spectroscopy. The laser powers between 80 and 800 mW were used. The formation of inner tubes was observed at a laser power of 160 mW. The amount of large diameter inner tubes (≈1 nm) increased with increasing laser power, whereas the smaller diameter tubes (≈0.5 nm) were destroyed. The inner tubes with intermediate diameter (≈0.7 nm) were stable at laser powers below 700 mW.

The authors of [144] investigated the growth properties of inner tubes inside SWCNTs filled with ferrocene and Pt (II) acetylacetonate molecules by Raman spectroscopy. It was found that the growth properties were strongly dependent on annealing temperature of the filled SWCNTs. The intensities of RBM peaks of inner tubes were significantly enhanced with increasing temperature. The inner tube growth depended on the type of metal catalyst. The inner nanotubes grew at higher temperatures with a Pt catalyst than with a Fe catalyst. It was observed that smaller diameter inner tubes were formed at lower annealing temperatures than larger diameter ones.

In [234], SWCNTs were filled with ferrocene and annealed at temperatures between 500 and 1000 °C. The analysis of the temperature and diameter-dependent growth of inner tubes was performed by multifrequency Raman spectroscopy. The growth temperatures of three individual-chirality inner tubes with chiralities of (6,5) (*d*_t_ = 0.753 nm), (14,1) (*d*_t_ = 1.142 nm) and (10,4) (*d*_t_ = 0.983 nm) were compared by tracing changes in the intensity of the tube RBM peaks with increasing annealing temperature [234]. The peak intensities of all of these inner tubes increased with increasing annealing temperature from 500 to 800 °C [234]. The different-diameter inner tubes were characterized by different growth rates. The larger diameter tubes had higher temperature of start of the growth. This temperature amounted to ≈500 °C for the 0.753 and 0.983 nm diameter tubes and 600 °C for the 1.142 nm diameter tubes. Also, the temperature at which the intensity of the inner tube peak was saturated increased from ≈700 °C for the 0.753 nm diameter tubes to ≈800 °C for the 1.142 nm diameter tubes [234].

**Figure 14:**
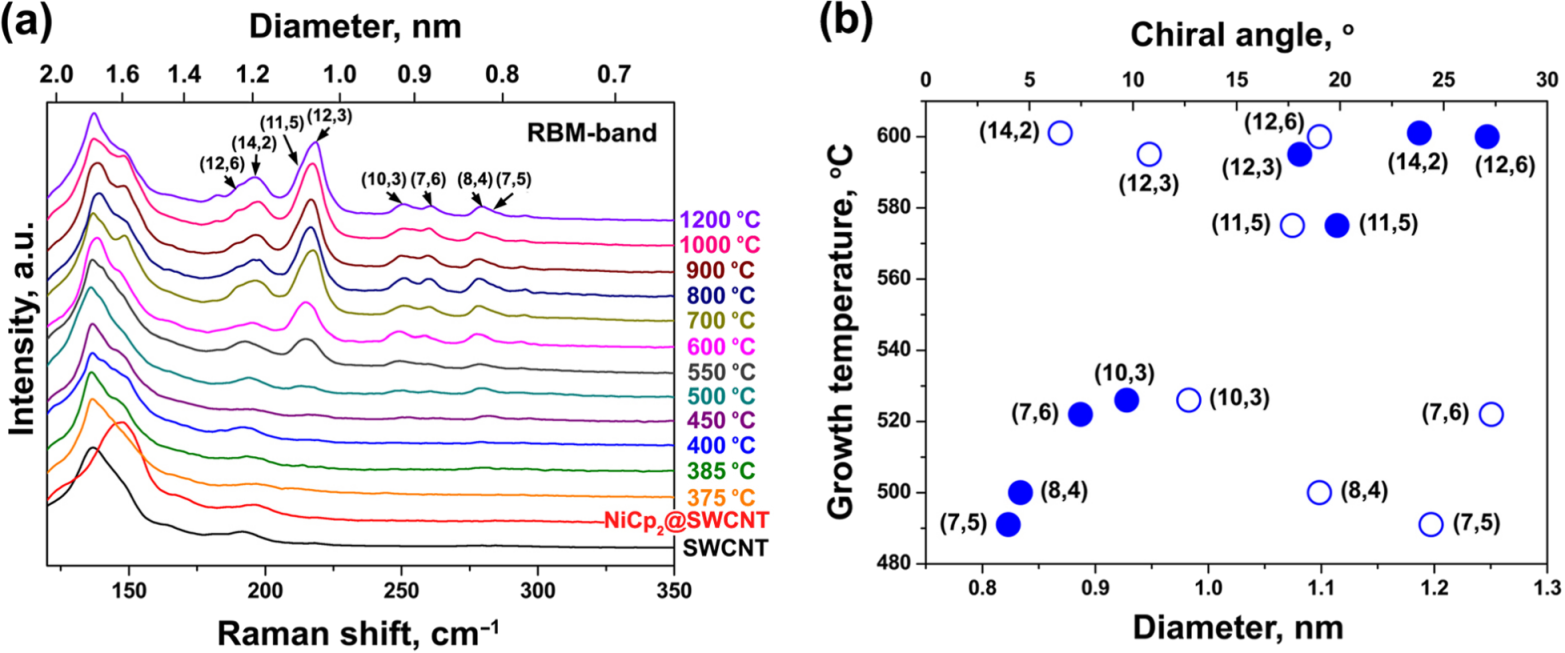
(a) The RBM-band of Raman spectra of the pristine, NiCp_2_-filled SWCNTs and samples annealed at temperatures between 375 and 1200 °C for 2 h acquired at a laser wavelength of 647 nm (*E*_ex_ = 1.92 eV). The chiral indexes of inner tubes are indicated above the respective peaks. (b) The temperature at which the intensity of inner tube RBM peak reaches half of its maximum plotted against the inner tube diameter (filled circles) and chiral angle (empty circles). The chirality indexes of the respective inner tubes are indicated near every circle. Reprinted with permission from [235], copyright 2015 Wiley-VCH Verlag GmbH & Co. KGaA, Weinheim.

The authors of [235] investigated the temperature-dependent inner tube growth inside the nickelocene-filled SWCNTs and samples annealed at temperatures ranging from 375 to 1200 °C. The changes in the intensity of the inner tube RBM peaks in Raman spectra of the annealed samples were traced (Figure 14a). For eight inner tubes with chiralities of (12,6), (14,2), (11,5), (12,3), (10,3), (7,6), (8,4) and (7,5) the temperature at which the intensity of inner tube RBM peak reaches half of its maximum was determined. This temperature was in the range from 490 to 600 °C for different inner tubes. Figure 14b shows the dependence of the growth temperature on the inner tube diameter and chiral angle [235]. It is clearly seen that the growth temperature is increased for larger diameter tubes. At the same time, the growth temperature does not depend on chiral angle of inner tubes [235].

## Conclusion

This paper presented the comprehensive review of the current status of research on growth dynamics of carbon nanotubes. The progress in synthesis methods of nanotubes, in particular the CVD approach, allowed obtaining high purity nanotube forests or individual tubes on substrates and even single chirality tubes, which is the key for the detailed investigation of their growth dynamics.

At present, vapor–liquid–solid and vapor–solid–solid models, the tip- and base-growth models as well as the tangential and perpendicular growth modes are well accepted for the growth of nanotubes. The authors debated whether the catalyst particle is in liquid or solid state during the nanotube growth. However, in situ HRTEM showed that the catalytic particles for SWCNTs and MWCNTs are solid at typical synthesis conditions. The still open questions are whether metallic catalyst particle does transform to carbide particle during the growth process, whether sub-surface intermediate carbide is formed on the metallic particle and whether the synthesis on purely metal carbide catalytic particle is possible. However, recent reports showed that typical catalysts undergo carburization (full or partial) under usual synthesis conditions of nanotubes and that metal carbides can catalyze the nanotube growth. The tuning of synthesis conditions of nanotubes allows obtaining the samples of single chirality tubes. However, the mechanism of chirality selective growth of nanotubes is still debated. The growth mechanism of inner tubes inside the host SWCNTs is different as compared to the mechanism of the nanotube growth in the CVD process. In the conventional bulk-scale synthesis, the nanotube growth terminates when the catalytic particle becomes deactivated by graphitic carbons shells. In contrast, the growth conditions of inner tubes inside the host SWCNTs are homogeneous and constant. The inner tube growth continues for many hours until the entire carbon source is exhausted.

Significant progress was achieved in the investigation of growth dynamics of carbon nanotubes. A mathematical growth model for description of catalyst lifetime-limited kinetics of the nanotube growth was obtained. It allowed quantifying the characteristics of growth dynamics, such as growth rate of nanotubes, lifetime of catalyst and activation energy of the tube growth. On the basis of the systematization and classification of the reports on the calculation of growth rates, the parameters on which the growth rate depends were highlighted. It was found that the growth rate of nanotubes depends on the pressure of carbon precursor, size and chemical nature of catalyst particle and synthesis temperature. On the basis of the systematization of the reports on the calculation of activation energy, the values characterizing the tube growth in the processes using various synthesis parameters (carbon precursor, catalyst, synthesis temperature, pressure) were classified. The assignment of the growth rate-limiting mechanisms on the basis of the calculated activation energies was analyzed. In many cases of the thermal CVD synthesis of nanotubes, bulk diffusion of carbon through the catalyst particle was found to be the process limiting the growth rate. In contrast, in the PECVD surface diffusion of carbon across the catalyst particle was the rate-limiting step. In some cases, contributions of multiple chemical processes were reflected in the activation energies, and the growth rate-limiting process changed during the synthesis procedure, depending on process parameters. The correlation between the growth rate of nanotubes and lifetime of the catalyst was revealed. The values were found to be inversely correlated: the lifetime increased while the growth rate decreased and vice versa. The systematization of the reports on the investigation of the growth properties of inner tubes inside SWCNTs filled with fullerene molecules showed that the nanotubes with a diameter close to that of C_60_ grew most rapidly, and the growth time increased with increasing the inner tube diameter. For the inner tubes formed inside SWCNTs filled with organometallic compounds, the growth temperatures were found to be higher for larger diameter tubes.

This review is a result of a detailed systematic investigation of 235 reports. It provides a valuable insight into growth dynamics of carbon nanotubes grown either in a CVD process or by nanochemical reactions inside host SWCNTs. The reports on the synthesis and investigation of nanotubes are for the first time summarized by the growth rates and calculated activation energies of the nanotube growth and growth rate-limiting steps. The conducted investigations allowed revealing the parameters on which growth dynamics of nanotubes depends, which opens a way of controlling the growth mechanism of nanotubes.

In conclusion, despite a large progress in the synthesis of nanotubes and understanding of their growth dynamics, many peculiarities of the growth mechanism are still debated. The synthesis of nanotubes in stable conditions with well-controllable synthesis parameters such as carbon precursor, catalyst, pressure and temperature is demanded for the production of nanotubes with well-defined properties.
